# Protonated nucleobases are not fully ionized in their chloride salt crystals and form metastable base pairs further stabilized by the surrounding anions

**DOI:** 10.1107/S2052252518006346

**Published:** 2018-06-08

**Authors:** Prashant Kumar, Malgorzata Katarzyna Cabaj, Aleksandra Pazio, Paulina Maria Dominiak

**Affiliations:** a Biological and Chemical Research Center, Department of Chemistry, University of Warsaw, ul. Żwirki i Wigury 101, Warszawa 02-089, Poland

**Keywords:** charge density, multipole refinement, quantum crystallography, electrostatic potential, electrostatic interaction energy, like-charged ions, nucleobases, cytosine, adenine, guanine, base pairs, intermolecular interactions, spin density, momentum density, hydrogen bonding, pharmaceutical solids, nucleic acid structures

## Abstract

This paper presents experimental charge-density studies of cytosinium chloride, adeninium chloride hemihydrate and guaninium dichloride crystals based on ultra-high-resolution X-ray diffraction data and extensive theoretical calculations. The results confirm that the cohesive energies of the studied systems are dominated by contributions from intermolecular electrostatic interactions, as expected for ionic crystals. Electrostatic interaction energies (*E*_es_) usually constitute 95% of the total interaction energy. The *E*_es_ energies in this study were several times larger in absolute value when compared, for example, with dimers of neutral nucleobases. However, they were not as large as some theoretical calculations have predicted. This was because the molecules appeared not to be fully ionized in the studied crystals. Apart from charge transfer from chlorine to the protonated nucleobases, small but visible charge redistribution within the nucleobase cations was observed. Some dimers of singly protonated bases in the studied crystals, namely a cytosinium–cytosinium *trans* sugar/sugar edge pair and an adeninium–adeninium *trans* Hoogsteen/Hoogsteen edge pair, exhibited attractive interactions (negative values of *E*_es_) or unusually low repulsion despite identical molecular charges. The pairs are metastable as a result of strong hydrogen bonding between bases which overcompensates the overall cation–cation repulsion, the latter being weakened due to charge transfer and molecular charge-density polarization.

## Abstract 2

Charge-density studies of cytosinium chloride, adeninium chloride hemihydrate and guaninium dichloride crystals based on ultra-high-resolution X-ray diffraction data and extensive theoretical calculations are presented. The studies confirm the importance of electrostatic interactions in ionic crystals and show how counterintuitive they can be for protonated nucleobases of like charge.

## 1. Introduction   

Nucleobases are naturally occurring chemical compounds that, together with phosphoric acid and sugars, constitute nucleic acids, the main information-carrying molecules of the cell which, by directing protein synthesis, determine the inherited characteristics of every living organism. However, they are also required for numerous other important functions within the cell, such as catalysing biological reactions or sensing and transmitting responses to cellular signals (Alberts *et al.*, 2002 ▸). Because of their crucial roles, nucleobases have been of considerable scientific interest since their discovery (Avery *et al.*, 1944 ▸; Watson & Crick, 1953*a* ▸,*b* ▸). Numerous reports concerning the noncovalent interactions of nucleobases (in general, hydrogen-bonding and π–π stacking interactions between bases, and cation–anion interactions with surrounding molecules) create an ever-expanding area of research in the scientific community (Cech, 1993*a* ▸,*b* ▸,*c* ▸; Cantor *et al.*, 1980 ▸). It is also now well established that nucleobases in nucleic acids are prone to forming not only canonical Watson–Crick pairs but also a variety of non-canonical pairs (Leontis & Westhof, 2001 ▸; Leontis *et al.*, 2002 ▸).

In the last two decades, the significance of the protonation (and deprotonation) of nucleobases for the biological functions of nucleic acids has been recognized (Halder *et al.*, 2015 ▸, 2014 ▸; Gehring *et al.*, 1993 ▸; Berger *et al.*, 1995 ▸; Chawla *et al.*, 2011 ▸). It was found that many nucleobases have elevated p*K*_a_ in nucleic acids and can be protonated at physiological pH (Acharya *et al.*, 2004 ▸; Goh *et al.*, 2013 ▸; Muth *et al.*, 2000 ▸; Pechlaner *et al.*, 2015 ▸; Siegfried *et al.*, 2010 ▸; Tang *et al.*, 2007 ▸; Ward *et al.*, 2014 ▸; Wilcox & Bevilacqua, 2013 ▸; Wolter *et al.*, 2017 ▸). For example, a near-neutral p*K*_a_ of 7.6 ± 0.2 was observed for a single adenosine in the ribosomal peptidyl transferase centre, which indicates a shift of ∼4.0 p*K*_a_ units from the unperturbed p*K*_a_ (Muth *et al.*, 2000 ▸).

Protonated nucleobases play an important role in defining three-dimensional structures, mediating conformational changes, controlling proper folding and stability, and facilitating catalysis (Bevilacqua *et al.*, 2004 ▸; Asensio *et al.*, 1998 ▸; Wilcox *et al.*, 2011 ▸). Changes in the protonation state, and resulting changes in local charge, have a significant influence on the propensity of nucleobases to form specific noncovalent interactions.

In the light of the above observations, we found it interesting to investigate the interactions of protonated bases with themselves and with anions surrounding them from a charge-density point of view. We focused our investigations on how nucleobases assemble themselves to form small-molecule crystals. In the present work, we show the results of our research on cytosinium chloride (CC), adeninium chloride hemihydrate (ACH) and guaninium dichloride (GDC) salts in the crystalline state for which it was possible to determine crystal charge densities experimentally. The crystal structures of CC, ACH and GDC were reported previously (Matković-Čalogović & Sanković, 1999 ▸; Broomhead, 1948 ▸; Cochran, 1951 ▸; Kistenmacher & Shigematsu, 1974 ▸; Cunane & Taylor, 1993 ▸; Mandel, 1977 ▸; Nieger, 2006 ▸). In addition, Cunane & Taylor (1993 ▸) published high-resolution X-ray diffraction data (sinθ/λ = 1.32 Å^−1^ at 123 K) for a single crystal of ACH. This first charge-density study mainly presented the refinement strategy and procedure of the studied system as well as its molecular electrostatic potential features, which was regarded as a great success at the time. For the other two, CC and GDC, only structural parameters have been reported until now.

The present paper is the first in a series of two. The series is dedicated to the qualitative and quantitative study of the interactions of the chosen protonated nucleobases with themselves, chlorine anions or water molecules by X-ray diffraction complemented with quantum mechanics calculations. Our aim is to find the relationship between molecular and crystal architecture, and analyse the nature of the intermolecular interactions. Here, we show detailed structural descriptions of the molecular motifs found in the studied crystals, to give a basis for further charge-density, electrostatic potential and interaction energy considerations. Then we focus on net molecular charges, chloride to protonated nucleobase charge transfer, molecular, dimer and crystal electrostatic potentials, and charge redistribution within nucleobases. We finish with an analysis of electrostatic interaction energies for dimers and whole crystals in order to relate, in a quantitative manner, the observed molecular electrostatic properties with the energies. Among the dimers analysed, in the present paper we focus on protonated nucleobase pairs. The next paper of the series will give a more detailed analysis of the interactions present in a variety of molecular dimers identified in the studied crystals. A comprehensive topological analysis of atom–atom intermolecular bonding and of the nature of electrostatic intermolecular interactions with respect to molecular and atomic multipole moments and charge-density penetration phenomena will be presented there. The analysis reveals how short-range (at single-atom, functional-group and whole-molecule levels) and long-range (at the level of second-nearest neighbours and further away) interactions contribute to the stability of the studied ionic crystals. The series aims to give a wide perspective on the electrostatic interactions which contribute to supramolecular assembly and thus on their crucial role in crystal engineering and, perhaps, structural biology.

## 2. Experimental and computational methods   

For a detailed description of the methods used, please see the supporting information.

### 2.1. Experimental charge-density models   

#### 2.1.1. Crystallization, data collection and processing   

The nucleobases were each added to a mixture of distilled water with a few drops of 38% HCl, and the mixtures were stirred and heated until the compounds had dissolved completely. The solutions were left to evaporate at room temperature in the case of cytosinium chloride (CC), and at 310 K in the cases of adeninium chloride hemihydrate (ACH) and guaninium dichloride (GDC). Crystals were obtained after one month.

For CC and ACH, single-crystal X-ray measurements were performed at 90 K on an Agilent Technologies SuperNova four-circle diffractometer equipped with a low-temperature nitro­gen gas-flow device (Oxford Cryosystems Cryostream Plus). For guaninium dichloride GDC, X-ray measurements were carried out on a Bruker APEXII ULTRA single-crystal diffractometer with a TXS rotating anode (Mo *K*α) equipped with a CCD-type area detector, multilayer optics and an Oxford Cryostream low-temperature attachment set to 100 K.

For CC and ACH, the determination of unit-cell parameters, integration of reflection intensities and data reduction, including multi-scan absorption corrections, were performed using *CrysAlis PRO* (Version 1.171.36.32; Agilent, 2013 ▸). Finally, reflection merging was carried out with the *SORTAV* program (Blessing, 1987 ▸, 1989 ▸, 1995 ▸, 1997 ▸). For GDC, the determination of unit-cell parameters, integration of reflection intensities and data reduction were performed with the *APEX2* suite of programs (integration was carried out with *SAINT*, Version 8.27B; Bruker, 2013 ▸), and the multi scan absorption correction, scaling and merging of reflection data were carried out with the *SORTAV* program.

#### 2.1.2. Structure solution and refinement   

Using *OLEX2* (Dolomanov *et al.*, 2009 ▸), the structures were solved with *SHELXS* (Sheldrick, 2008 ▸, 2015 ▸) with direct methods and refined with olex2.refine using the independent-atom model (IAM).

Multipole refinements were performed in *MoPro* (Guillot *et al.*, 2001 ▸; Jelsch *et al.*, 2005 ▸) with the use of the Stewart–Hansen–Coppens multipolar model (Stewart *et al.*, 1975 ▸; Hansen & Coppens, 1978 ▸). Refinements were performed against structure factor amplitudes (*F*) with the *I* ≥ 2σ(*I*) cut-off. The initial atomic coordinates, *x*, *y* and *z*, for all atoms, the anisotropic atomic displacement parameters (*U_ij_*) for non-hydrogen atoms and the isotropic atomic displacement parameters for hydrogen atoms were taken from the IAM refinement. Local Cartesian coordinate systems and the initial multipolar and contraction–expansion parameters for nucleobases and water molecules were defined by *LSDB* (Volkov, Li *et al.*, 2004 ▸) combined with *UBDB2011* (Jarzembska & Dominiak, 2012 ▸). Each atom was assigned core and spherical valence scattering factors derived from the Su & Coppens (1998 ▸) atomic wavefunctions for neutral-atom configurations, except for the chlorine atoms. For chlorine atoms two possibilities were investigated: the Cl^−1^ ion scattering radial function and ion electronic configuration, or the Cl^0^ neutral scattering radial function and neutral electronic configuration. The anomalous dispersion coefficients were taken from Kissel *et al.* (1995 ▸). The contraction–expansion parameters, κ and κ′, for all hydrogen atoms were kept fixed at the *UBDB2011* values during refinement. The κ′ parameters of the chlorine atoms were restrained to 1.0. The values of the *U_ij_* parameters for the hydrogen atoms were estimated from the *SHADE 3.0* server (Madsen, 2006 ▸). The *X*—H distances were restrained to the average values obtained from neutron diffraction studies (Allen & Bruno, 2010 ▸) with a restraint σ of 0.004 Å. As atom Cl1 of ACH undergoes noticeable an­harmonic motion, Gram–Charlier (GC) coefficients (Kuhs, 1983 ▸; Johnson, 1969 ▸; Scheringer, 1985 ▸) up to the third order were used to model it, while the physical reliability of the anharmonic model was confirmed by the probability density function computed with *MolIso* (Hübschle & Luger, 2006 ▸).

**Figure 1:**
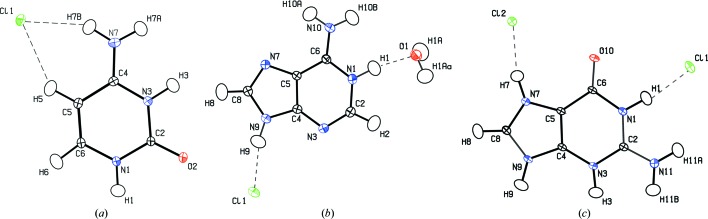
*PLATON* drawings, showing 50% probability ellipsoids, for the cytosinium chloride (CC), adeninium chloride hemihydrate (ACH) and guaninium dichloride (GDC) crystals.

The outcomes of the multipole refinements were verified by examining the *R* factors, goodness of fit (GOF) and residual densities (Table 1 ▸). The evaluation was additionally supported by *JNK2RDA* (Meindl & Henn, 2008 ▸) and *XDRKplot* implemented in the *WinGX* package (Farrugia, 2012 ▸) (see Figs. S1–S4 in the supporting information). *PLATON* diagrams (Spek, 2009 ▸) with the atom-labelling schemes are shown in Fig. 1 ▸. The CIF files can be retrieved from the Cambridge Structural Database (CSD) (Groom *et al.*, 2016 ▸) (deposition numbers 1539172–1539174). Sets of raw diffraction frames and the associated data are accessible online under the following DOIs: https://doi.org/10.18150/repod.7313736, https://doi.org/10.18150/repod.0481200 and https://doi.org/10.18150/repod.8020589 (Repository for Open Data, ICM, University of Warsaw, Poland).

### 2.2. *UBDB* charge-density models   

*UBDB* models of charge densities for CC, ACH and GDC crystals were built on experimental geometries using *LSDB* (Volkov, Li *et al.*, 2004 ▸) and *UBDB2011* (Jarzembska & Dominiak, 2012 ▸). Chlorine atoms were treated as spherical anions with a Cl^−1^ ion scattering radial function and ion electronic configuration. Core and spherical valence factors for each atom were taken from Su and Coppens’ atomic wavefunctions (Su & Coppens, 1998 ▸). For the majority of calculations, the net charge of each molecule was scaled separately to its formal value: +1 e for cytosinium and adeninium, +2 e for guaninium, 0 e for water molecules and −1 e for chloride anions. For alternative simulations (as explained further in the text), net molecular charges were scaled to experimentally observed values.

### 2.3. Periodic quantum mechanical calculations   

Periodic quantum mechanical calculations were applied to compute theoretical crystal charge densities, theoretical structure factors and crystal cohesive energies (*E*_coh_). The *CRYSTAL14* package (Dovesi, Orlando *et al.*, 2014 ▸; Dovesi, Saunders *et al.*, 2014 ▸) was used at the DFT-B3LYP/pVDZ level of theory (Becke, 1988 ▸; Perdew, 1986 ▸; Lee *et al.*, 1988 ▸; Dunning, 1989 ▸). The computations were done in two versions, for experimental and optimized geometries. During optimization, the cell parameters were fixed while the atom positions were allowed to vary. The Grimme dispersion correction (Grimme, 2006 ▸) was applied for all calculations, whereas a correction for basis set superposition error (BSSE) (Civalleri *et al.*, 2008 ▸) was applied for the computation of cohesive energy following the supermolecular approach (Maschio *et al.*, 2011 ▸). The irreducible Brillouin zone was sampled using 170 *k* points (the shrinking factor of the reciprocal space net was set to 8). See Table S1 for further details. Static theoretical structure factors were computed up to sinθ/λ = 1.30 Å^−1^ using the XFAC option in *CRYSTAL14* (Flack, 1984 ▸; Le Page & Gabe, 1979 ▸). Multipole refinements against theoretical structure factors were performed using *MoPro* (Guillot *et al.*, 2001 ▸; Jelsch *et al.*, 2005 ▸) with a strategy analogous to that of the experimental structure factors. The scale factors and the *x*, *y* and *z* parameters were not refined. Basic statistical descriptors of the refinement are given in Table 2 ▸ and more information can be found in the supporting information (see text and Figs. S5–S12 and S14).

### 2.4. Hirshfeld surface and QTAIM analyses   

Hirshfeld surface analyses (Spackman & McKinnon, 2002 ▸; McKinnon *et al.*, 2007 ▸) were performed for experimental geometries using *Crystal Explorer* (Version 3.3; Wolff *et al.*, 2012 ▸). Wavefunction calculations were done for each molecule separately, applying their formal charge, at the HF/6-31G** level (Hehre *et al.*, 1972 ▸) using *GAUSSIAN*09 (Frisch *et al.*, 2016 ▸).

A QTAIM (quantum theory of atoms in molecules; Bader, 1990 ▸) analysis was carried out on all electron-density models of CC, ACH and GDC crystals. For models in the multipole representation (experimental, *UBDB* and the one fitted to the theoretical structure factors), integrated charges were computed using the *WinXPRO* program (Stash & Tsirelson, 2002 ▸, 2007 ▸). For exact (not approximated by the multipolar model) theoretical crystal electron densities, integrated charges were calculated for experimental and optimized geometries using *TOPOND14* (Gatti *et al.*, 1994 ▸; Tsirelson, 2002 ▸).

### 2.5. Electrostatic potentials   

Electrostatic potentials for single molecules, selected dimers and entire crystals were computed from charge-density models in multipole representation with the use of the *XDPROP* module of *XD2016* (Volkov *et al.*, 2016 ▸) and visualized by *MoleCoolQt* (Hübschle & Dittrich, 2011 ▸).

### 2.6. Intermolecular interaction energies and electrostatic contributions to them   

To compute intermolecular interaction energies for isolated dimers with geometries as found in the studied crystals, the density functional theory-based symmetry-adapted perturbation theory (DFT-SAPT) (Jansen & Hesselmann, 2001 ▸; Williams & Chabalowski, 2001 ▸; Jeziorski *et al.*, 1994 ▸) method was applied. Formal charges were assigned to particular molecules. The total intermolecular interaction energy in the DFT-SAPT method is given as the sum of the first- (*E* ^1^) and second-order (*E* ^2^) perturbation energy terms and the 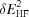 energy term, specifically electrostatic (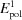), induction (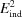) and dispersion (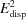) energy terms, together with exchange-repulsion (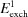, 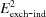 and 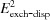) terms: 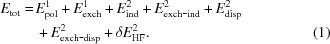
The correction 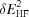 was applied in all cases to estimate the polarization effect beyond the second order. The DFT-SAPT calculations applied Kohn–Sham (KS) orbitals which were determined using the PBE0AC exchange-correlation potential (Heßelmann & Jansen, 2002 ▸; Gross *et al.*, 1996 ▸). For neutral and cationic molecules, the asymptotic behaviour of the functional was corrected. The values of the highest occupied molecular orbital (HOMO) and ionization energies were calculated using the PBE0 functional. The anionic systems (chloride anions) were left without asymptotic correction (Lee & Burke, 2010 ▸). All DFT-SAPT and HOMO energy calculations were done in *MOLPRO2012.1* (Werner *et al.*, 2012 ▸) with the aug-cc-pVTZ Dunning basis set (Kendall *et al.*, 1992 ▸; Dunning, 1989 ▸).

For the calculation of electrostatic, polarization, dispersion and repulsion contributions to the lattice energy, the semi-classical density sum (the *PIXEL* method; Gavezzotti, 2011 ▸) was used, which relies on a dimer approximation. Molecular electron densities of molecules bearing their formal charges were obtained using *GAUSSIAN09* at the MP2/6-31** level (Hariharan & Pople, 1973 ▸). The electron densities were then analysed using the *PIXELc* module (Gavezzotti, 2003*a* ▸,*b* ▸,*c* ▸,*d* ▸) of the Coulomb–London–Pauli (*CLP*) program (Gavezzotti, 2011 ▸) which allows the calculation of lattice energies. The total intermolecular interaction energy was defined in *PIXEL* as follows 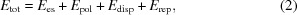
where *E*_es_ is the electrostatic interaction energy, *E*_pol_ is the polarization energy, *E*_disp_ is the dispersion energy and *E*_rep_ is the repulsion energy between interacting molecules. Due to the limitations of the program, *i.e.* a maximum of two mol­ecules are allowed in the asymmetric unit, the crystal cohesive energy was only computed for CC.

Electrostatic contributions to intermolecular interaction energies and to crystal cohesive energies were also computed from experimental, *UBDB* and theoretical multipolar models of charge densities with the *XDPROP* module of the *XD2016* package (Volkov *et al.*, 2016 ▸). The exact electrostatic energy (*E*_es_) was computed with the use of the EPMM method (Volkov, Koritsanszky & Coppens, 2004 ▸). The electrostatic energies from molecular monopole moments (point charges, *E*_Coul_) were obtained by extracting monopole–monopole contributions from calculations with the mMM option or, alternatively, computed directly from Coulomb’s law, *E*_Coul_ = *q*_1_ *q*_2_/4π∊_0_ *r*. To differentiate between results for dimers and results for the whole crystal, energies for the latter will be abbreviated as *E*_coh,es_ and *E*_coh,Coul_.

It is to be noted that 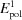 from equation (1) refers to the exact electrostatic interaction energy and from now onwards will be abbreviated as *E*_es_.

### 2.7. Transition-state search   

For two selected dimers of the protonated nucleobases, a transition-state search was performed in *GAUSSIAN16* (Frisch *et al.*, 2016 ▸). All the geometry optimizations, transition-state searches and vibrational frequency analyses were performed with the hybrid B3LYP functional using the 6-311G(d,p) basis set. The B3LYP method has repeatedly been shown to yield results that are at least equal to MP2 calculations (Singleton *et al.*, 1997 ▸). To describe dispersion interactions properly, the Becke–Johnson damping function approach DFT-D3(BJ) was used (Grimme *et al.*, 2011 ▸).

## 3. Results and discussion   

### 3.1. Packing and structural details   

Crystal structures based on conventional crystal structure analyses of all three samples have been reported previously (Broomhead, 1948 ▸; Cochran, 1951 ▸; Kistenmacher & Shigematsu, 1974 ▸; Cunane & Taylor, 1993 ▸; Matković-Čalogović & Sanković, 1999 ▸; Mandel, 1977 ▸; Nieger, 2006 ▸). Here, we will repeat some of the previously discussed aspects of the structures and point to some new ones, in order to compare all three samples and give a structural basis for further discussion of the interactions in terms of electron density and energy.

**Figure 2:**
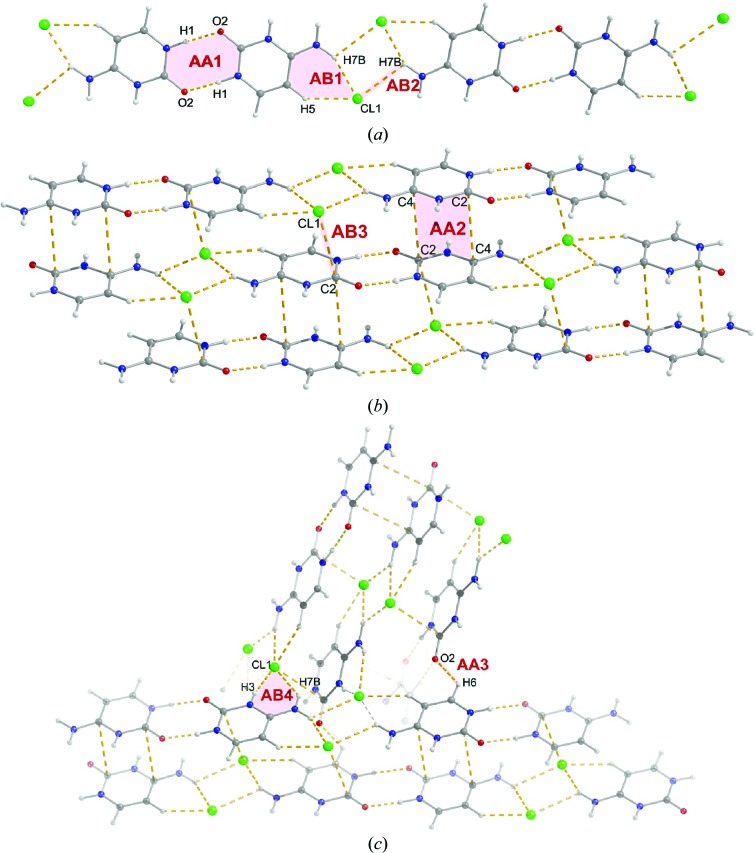
Dimeric motifs in the cytosinium chloride (CC) crystal.

Cytosinium chloride (CC) crystallizes in the monoclinic space group *P*2_1_/*n* with one 1*H*,3*H*-cytosinium cation and one chloride anion in the asymmetric unit. Despite their presumably overall repulsive interaction due to their formal charges, the two cytosinium cations are arranged into a centrosymmetric *trans* sugar/sugar edge base pair linked by two N1—H1⋯O2 hydrogen bonds [see dimer AA1 in Fig. 2 ▸(*a*), Table 3 ▸ for geometric information, and Fig. S15 for nucleobase edge nomenclature conventions (Leontis & Westhof, 2001 ▸; Leontis *et al.*, 2002 ▸)]. By the term ‘base pair’ we mean dimers of bases in which the bases lie roughly in the same plane and there are at least two hydrogen bonds (classical and/or weak) between the bases. The second possible base pair to be formed by two cytosinium cations, in which two N3—H3⋯O2 hydrogen bonds could develop to create a *trans* Watson–Crick/Watson–Crick edge base pair, is not observed here in the CC structure. Instead, the Watson–Crick and Hoogsteen edges of the cytosinium cations are involved in direct interactions with the chloride anions.

The AA1 dimers form an infinite one-dimensional ribbon, held together by two chloride anions interacting with the Hoogsteen edges of the cytosinium cations through N7—H7*B*⋯Cl1 and C5—H5⋯Cl1 hydrogen bonds on one side of the chloride anions (dimers AB1) and through an N7—H7*B*⋯Cl1 bond on the other side of the anions (dimers AB2, Fig. 2 ▸ *a*). These ribbons further stack together to form a layer, shown in Fig. 2 ▸(*b*). Inside a single stack, the ribbons are shifted by *ca* 4.3 Å along the ribbons and towards one of the edges by *ca* 1.2 Å, with a distance between two ribbons of 3.375 Å. The relative orientation of the ribbons in a stack is probably related to optimizing the distribution of molecules of opposite charge: the centre of a cytosinium cation in one ribbon lies almost exactly above a chloride anion in another ribbon, thus allowing for π⋯Cl^–^contacts (dimers AB3) (Schottel *et al.*, 2008 ▸; Mooibroek *et al.*, 2008 ▸; Patrick, 2014 ▸; Chifotides & Dunbar, 2013 ▸). In addition, repulsive cation–cation interactions between ribbons are, presumably, partially counterbalanced by stacking interactions between two cytosinium rings (dimers AA2).

Two adjacent layers of ribbon stacks interact with each other in such a way that each chloride anion is in close contact through N7—H7*A*⋯Cl1 and N3—H3⋯Cl1 hydrogen bonds with yet another cytosinium cation (dimer AB4, see Fig. 2 ▸ *c*). The N3—H3⋯Cl1 hydrogen bond is the shortest among all of the N—H⋯Cl hydrogen bonds observed in the CC structure and is generally among the shortest N—H⋯Cl bonds found in the CSD (Steiner, 1998 ▸; Mascal, 1997 ▸). The cations of one stack, and the ribbons to which they belong, are tilted by *ca* 57° with respect to the cations (and their ribbons) of another stack. In addition to cation–anion interactions between the layers of the stacks, cytosinium cations from two adjacent layers are in direct contact with each other through C6—H6⋯O2 hydrogen bonds (dimer AA3).

**Figure 3:**
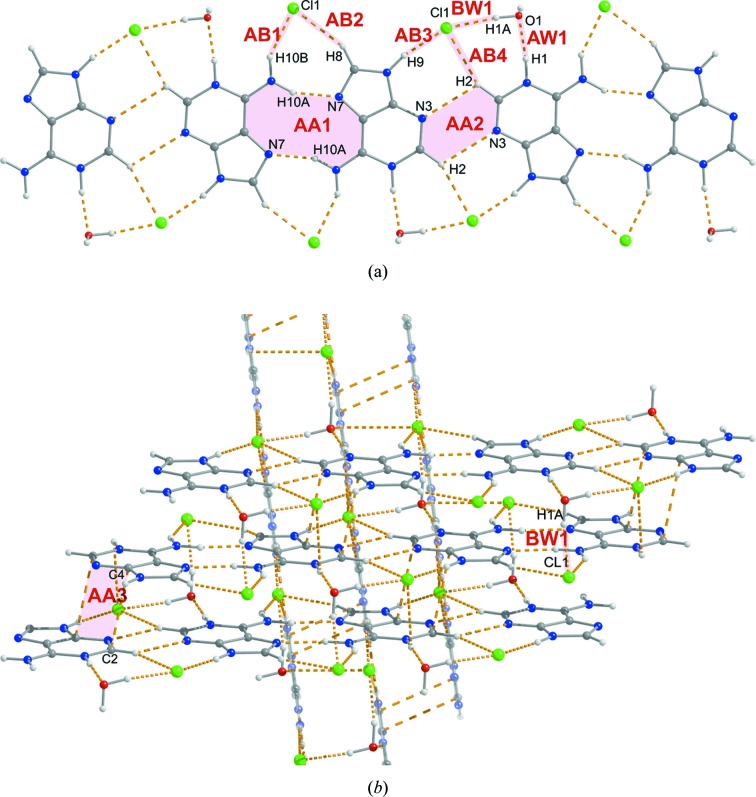
Dimeric motifs in the adeninium chloride hemihydrate (ACH) crystal.

Adeninium chloride hemihydrate (ACH) crystallizes in the space group *P*2/*n*, with the asymmetric unit consisting of one adeninium cation, one chloride anion and one half of a water molecule located on a twofold rotation axis. The cation adopts the most common 1*H*,9*H*-adeninium tautomer (Marian *et al.*, 2005 ▸). From the variety of bidentate hydrogen-bonded base pairs which could be formed by adeninium cations, only two are observed in the structure of ACH: a centrosymmetric *trans* Hoogsteen/Hoogsteen edge pair with two N10—H10*A*⋯N7 hydrogen bonds (dimer AA1) and a centrosymmetric *trans* sugar/sugar edge pair with two C2—H2⋯N3 hydrogen bonds (dimer AA2, Fig. 3 ▸ *a*). Atom C8 from the Hoogsteen edge, atom N9 from the sugar edge and the whole Watson–Crick edge are exposed to chloride anions and water molecules.

Both adeninium base pairs are arranged together into infinite one-dimensional ribbons (Fig. 3 ▸ *a*) where the ribbon edges are framed by chloride ions and water molecules. Each AA1 dimer of cations is embraced by two chloride anions through N10—H10*B*⋯Cl1 and C8—H8⋯Cl1 hydrogen bonds (dimers AB1 and AB2, respectively), while each AA2 dimer is surrounded by two further chloride ions through N9—H9⋯Cl1 and C2—H2⋯Cl1 hydrogen bonds (dimers AB3 and AB4, respectively). To satisfy the hydrogen donor remaining at the Watson–Crick edge of the adeninium cation, a water molecule is positioned to form an N1—H1⋯O1 hydrogen bond (dimer AW1). Additionally, the oxygen atom donates a proton to the aforementioned chloride anions to form an O1—H1*A*⋯Cl1 hydrogen bond (dimer BW1). According to the geometric data, the O1—H1*A*⋯Cl1 and N9—H9⋯Cl1 hydrogen bonds seem to be the strongest hydrogen bonds involving chloride ions in the ACH structure.

Similarly to the CC structure, ribbons in the ACH crystal stack together to form two-dimensional layers (Fig. 3 ▸ *b*). Stacked ribbons are shifted along by almost an entire mol­ecule (*ca* 2.95 Å) and very slightly (*ca* 0.5 Å) towards one of the edges. The distance between the ribbons is *ca* 3.19 Å. For ACH, the relative shift of the ribbons is probably associated with minimization of anion–anion and cation–cation repulsion. Chloride anions of adjacent ribbons are placed above and below the empty space between the chloride anions or above and below the water molecule of the central ribbon. The water molecule forms a second O1—H1*A*⋯Cl1 hydrogen bond (symmetry-related to BW1 mentioned above) with one of the chloride ions of an adjacent ribbon. For adeninium cations from adjacent ribbons, only close contacts between atoms on the sugar edges were observed (dimers AA3).

Again, as in the case of the CC structure, adjacent stacks of ribbons are connected to each other by chloride and water molecules, here a common component in both interacting stacks (Fig. 3 ▸ *b*). Thus, no new types of dimer are formed, albeit each chloride ion involved in dimers AB1 and AB2 in one ribbon forms dimers AB3, AB4 and BW1 with a ribbon from an adjacent layer of stacks. The ribbons from two adjacent stacks are tilted with respect to each other by *ca* 83°. There is no direct contact between cations from neighbouring layers of stacks.

For guaninium dichloride (GDC), the asymmetric unit in the orthorhombic space group *Pnma* consists of one half of a guaninium dication and two halves of chloride anions. All atoms of GDC lie in a special position on the crystallographic mirror plane. All heterocyclic nitrogen atoms are protonated in the guaninium dication.

**Figure 4:**
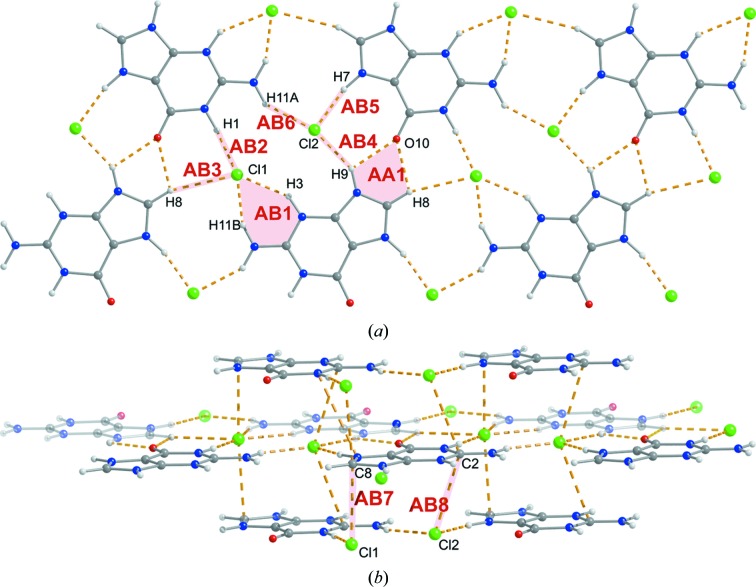
Dimeric motifs in the guaninium dichloride (GDC) crystal.

Neither of the two possible bidentate hydrogen-bonded base pairs of guaninium dications is present in the structure of GDC. Contrary to the two previously discussed structures, there is no ribbon architecture observed here. Instead, two-dimensional layers of guaninium dications and chloride anions being in contact with each other through hydrogen bonds (Fig. 4 ▸ *a*) are present. Each chloride anion in the layer is in direct hydrogen-bonding contact with three guaninium dications. For the Cl1 ion, the N11—H11*B*⋯Cl1 and N3—H3⋯Cl1 hydrogen bonds are present in dimer AB1, N1—H1⋯Cl1 in dimer AB2 and C8—H8⋯Cl1 in dimer AB3. For the Cl2 ion, the hydrogen bonds are N9—H9⋯Cl2 in dimer AB4, N7—H7⋯Cl2 in dimer AB5 and N11—H11A⋯Cl2 in dimer AB6. Among the N—H⋯Cl hydrogen bonds, N1—H1⋯Cl1 and N7—H7⋯Cl2 seem to be the strongest ones. The remaining close contacts within the single layer are between two guanine dications through N9—H9⋯O10 and C8—H8⋯O10 hydrogen bonds (dimer AA1). The layers of ions stack with each other in such a way that the Cl1 anions are almost exactly below and above atom C8 of the five-membered ring of the guaninium dication (dimers AB7), and the Cl2 anions are almost exactly below and above the six-membered ring (dimers AB8) (Fig. 4 ▸ *b*). There are no close atom–atom contacts indicating stacking interactions between the guaninium dications. The distance between the layers is 3.242 Å.

**Figure 5:**
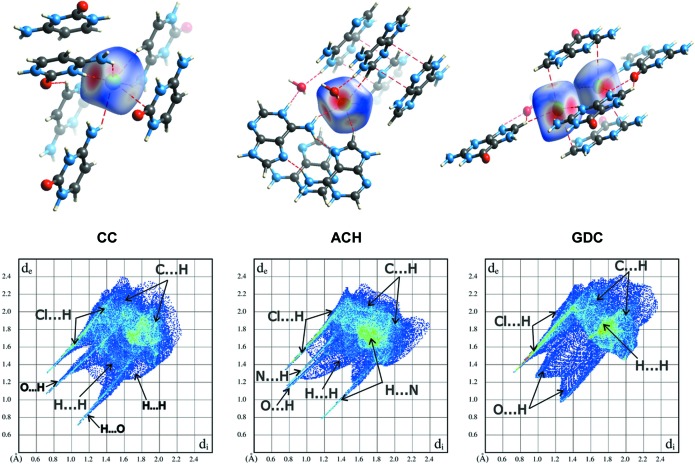
(Top) *d*_norm_ mapped on a Hirshfeld surface of the chloride anions to visualize the contacts with surrounding molecules, and (bottom) fingerprint plots of the nucleobase cations, in (left) CC, (middle) ACH and (right) GDC crystals.

To understand the architecture of these crystals further, it is essential to take a closer look at the overall mol­ecular packing features. The Hirshfeld surface is a useful tool to investigate detailed aspects of molecular packing. First of all it allows identification of all molecules which are in direct contact with the molecule under consideration, regardless of whether they are close enough to be found using the van der Waals radii concept or not. After computing the Hirshfeld surfaces for each molecule separately in the studied structures, it appears that the molecules are surrounded by more close neighbours than have been discussed until now. For example, in the case of the chloride anion in CC (Fig. 5 ▸), six cytosinium cations, not four, contribute to its Hirshfeld surface. The *d*_norm_ function mapped on the Hirshfeld surface nicely shows several red spots, indicating very close intermolecular interactions. However, it is obvious from the fingerprint plots associated with each Hirshfeld surface (Fig. 5 ▸ and Fig. S16) that, besides atom–atom contacts which fall below the van der Waals limit and manifest on the plots as sharp spikes, there are more intermolecular atom–atom contacts present in these structures. Analysis of the percentage contribution of the observed contacts to the Hirshfeld surfaces (Fig. S17) confirms that the previously identified hydrogen bonds of *D*—H⋯Cl^−^ and *D*—H⋯N/O types are very common in all the studied structures. Also, *D*—H⋯(C)π contacts are seen in all three structures. Not only previously identified (C)π⋯Cl^−^ contacts, but also (N)π⋯Cl^−^ contacts appear in the studied crystals, mainly in CC and GDC. In addition to the known CC and ACH (C)π⋯(C)π contacts, ones involving nitrogen atoms also appear in these two structures. What is qualitatively new with respect to atom–atom contacts identified on the basis of van der Waals radii (Table 3 ▸) is that the Hirshfeld surface analysis shows that H⋯H contacts between nucleobases are the most common in these crystals. This suggests their importance in crystal structure stabilization. It should also be noted that, from a chloride anion point of view, the CC and GDC structures are more similar to each other than to ACH. In the latter, intermolecular interactions between chloride ions are facilitated almost exclusively by hydrogen bonds, whereas in the former, (C)π⋯Cl^−^ interactions also seem to be important.

### 3.2. Molecular charges   

**Figure 6:**
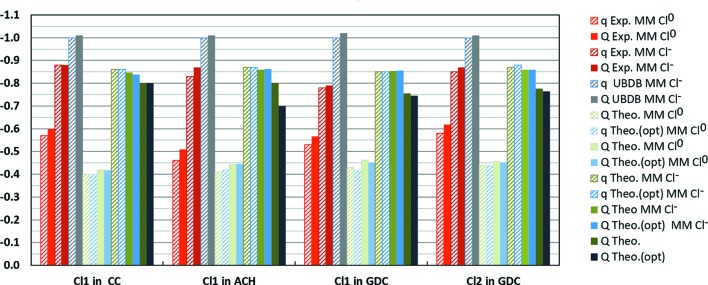
Values of chloride anion charges (e) in studied crystals according to different methods: q – charge computed directly from *P*_val_ parameters of the multipolar model (MM) obtained from *UBDB* modelling (*UBDB*) or from refinements against either experimental (Exp.) or theoretical (Theo.) structure factors with the use of ionic (Cl^−^) or neutral (Cl^0^) radial scattering functions for chloride; Q – charge computed from integration over atomic basins of crystal electron densities from various multipolar models or directly from periodic quantum mechanical calculations. (opt) – results from periodic calculations with geometry optimization.

The studied crystals are ionic in nature, so for them in particular electrostatic interactions are expected to play a major role in crystal stability. One of the most important factors when analysing intermolecular interactions is mol­ecular charge. According to the experimental charge densities of the studied crystals, the charges on the nucleobase and chloride ions are not equal to the formal ones, *i.e.* the mol­ecules are not fully ionized, as was already suggested for ACH by Cunane & Taylor (1993 ▸). Some degree of electron-density transfer from the chloride ions to the protonated nucleobase cations was observed. In all the studied structures, the chloride anions bear less negative charge, between −0.78 (4) e and −0.88 (5) e as computed directly from the *P*_val_ parameters of the multipolar model (Fig. 6 ▸). Accordingly, the nucleobase ions are less positively charged. Experimental charge-density integration over atomic basins calculated using QTAIM analysis leads to similar values of molecular charge. Moreover, topological charges computed directly from periodic calculations provide independent support to the finding that the chloride anions are less negative in these crystals; their charges are in the range of −0.70 e to −0.80 e according to theory. Values of charge departing from the formal −1 e for chloride anions in organic salts have recently been reported (Niranjana Devi *et al.*, 2017 ▸; Wang *et al.*, 2017 ▸; Nelyubina & Lyssenko, 2015 ▸; Nelyubina *et al.*, 2015*a* ▸,*b* ▸, 2007 ▸).

Interestingly, the charge on the water molecule in ACH equals almost 0.0 e according to both experiment and theory.

In order to gain more evidence supporting our findings regarding charge transfer, we decided to compare experimental charge-density models with those in multipolar model representations simulated from periodic quantum mechanical calculations. On the basis of the periodic calculations, we computed static theoretical structure factors and fitted multipolar models to them. In addition, we checked how usage of the Cl^0^ neutral scattering radial functions instead of Cl^−1^ ionic functions influences the results.

First of all, it appears that multipolar models fitted in reciprocal space to the theoretical charge densities reproduce the molecular topological charges very well, despite the known limitations of multipolar models in describing electron densities in the core region (Fischer *et al.*, 2011 ▸), as visible on residual maps (see the text and Figs. S5, S7, S9, S11 and S14 in the supporting information). Molecular topological charges computed from multipolar models differ by only *ca* 0.08 e from the target ones and are always too negative for chloride ions (and too positive for nucleobase cations).

The above is true only if Cl^−1^ ionic scattering radial functions are used. When Cl^0^ neutral scattering radial functions are used, the charge on the chloride ions in the multipolar model representation is substantially reduced, up to *ca* −0.42 e, and correspondingly the same happens for the molecular charges of the appropriate nucleobases. However, from *R*-factor statistics and residual-density analysis (Table 2 ▸, and Fig. S5–S12) it can be concluded that, in the case of theoretical charge densities, Cl^−1^ ionic scattering radial functions are more appropriate. They allow refinement of multipolar models which provide charge densities closer to the target ones, yet they slightly overestimate the absolute values of the molecular charges.

There is no substantial difference in all the above findings when periodic calculations performed for experimental geometries are compared with calculations that include a geometry optimization step. Apparently, the differences in hydrogen-atom positions (the *X*—H distances differ the most among covalent bonds when these two geometries are compared) and the fact that only the latter calculations are based on systems in equilibrium do not matter here; the experimental geometry is accurate enough.

In every case, topological molecular charges computed from multipolar models are very similar (*ca* ±0.02 e) to those derived directly from *P*_val_. This proves that multipolar models of particular molecules are well behaved and do not show too much delocalization compared with molecules of another kind. This is important for further intermolecular interaction energy calculations.

In the case of the experimental data, the use of Cl^0^ neutral scattering radial functions also leads to a reduction in the molecular charge from the formal one, but the reduction is not as large as in the case of the theoretical simulations. For all three structures, the chloride anions bear charges between −0.46 (4) e and −0.58 (5) e as computed directly from the *P*_val_ parameters of the multipolar model (Fig. 6 ▸). In contrast with the theoretical simulations, refinement statistics (*R* factors, statistical plots and residual-density analysis; Table 1 ▸ and Figs. S1–S4) are slightly better for models with neutral scattering factors than with ionic, but the difference is not large enough to disregard (because of, for example, possible functional or basis-set limitations; Medvedev *et al.*, 2017 ▸; Cutini *et al.*, 2016 ▸) conclusions resulting from the theoretical simulations or to oppose the principle of Ockham’s razor: upon crystal formation, chloride anions most probably retain their ionic nature and they are still more ionic than neutral. Thus, we decided that multipolar models using Cl^−1^ ionic scattering radial functions are the best models of charge density which can be achieved from the fits to the collected experimental diffraction data of the studied crystals.

To summarize, in our opinion it is reasonably well proven by the data presented here that the chloride ions in the studied crystals are not fully ionized, and accordingly the same holds for the protonated nucleobases. The phenomenon might be general for all possible nucleobase chlorides and some other organic hydrochloride salts.

The theoretical simulations also helped to increase understanding of the possible causes of residual electron density observed around chloride anions in the case of the experimental data. On residual maps from the theoretical simulations there are no such pronounced residual features close to the chloride-ion positions that are visible on the experimental maps (Fig. S13). Thus, the assumption that experimental static electron densities are properly modelled by a multipolar model is justified. The features might be attributed either to anharmonic motion or to experimental errors like, for example, poor absorption corrections. Indeed, refinement of the third-order anharmonic parameters in the case of ACH (the only data set with a resolution high enough to justify the refinement) clears the residual plots a lot (Figs. S1 and S13).

### 3.3. Electrostatic potential of molecules and crystals   

The complementarity of molecular electrostatic potentials is believed to be one of the most important factors influencing how molecules interact with each other (Politzer & Murray, 2015 ▸).

**Figure 7:**
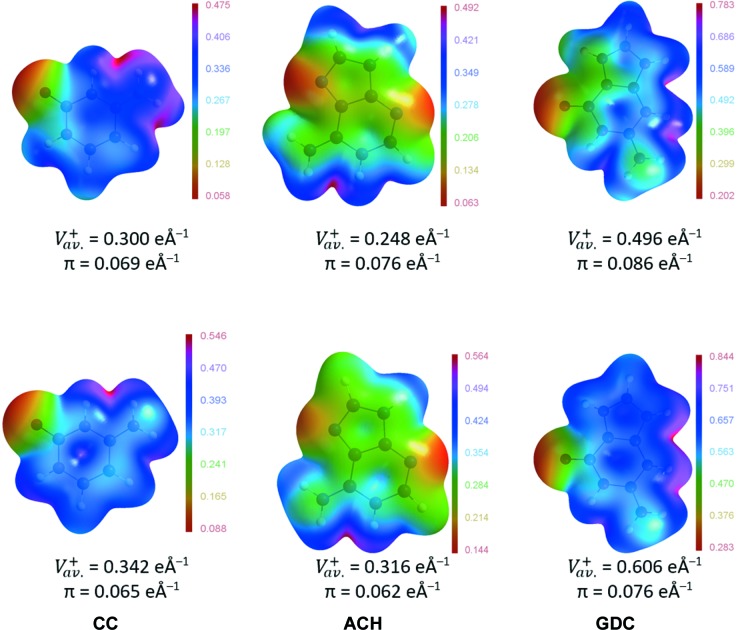
Electrostatic potentials (e Å^−1^) of (top) nucleobases in the studied crystals from experimental charge densities or (bottom) isolated nucleobases bearing formal charges as modelled by *UBDB*, mapped on the respective electron-density isosurfaces at ρ = 0.0135 e Å^−3^ (= 0.002 a.u.). 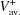 is the average of the positive surface values and π is the average deviation from the average positive surface value (Politzer *et al.*, 2001 ▸).

In the case of ionic molecules, complementarity of mol­ecular charge, *i.e.* positive charge complementing negative and *vice versa*, is an obvious observation. But in the crystals studied here, direct contacts between molecules with like charges, *i.e.* the protonated nucleobases, are observed. Interestingly for this type of molecular contact, some degree of complementarity can be observed (Fig. 7 ▸ top, and Figs. 2 ▸ and 3 ▸). Moreover, the electrostatic potential at the molecular surface for molecules in a crystal differs noticeably from that of isolated molecules (Fig. 7 ▸ top and bottom, and Fig. S17). The values of the electrostatic potentials of the former are, obviously, shifted towards less positive ones due to the fact that molecules in a crystal are less positive than isolated molecules with formal charges assigned to them (see the values of 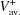 in Fig. 7 ▸). However, the electrostatic potential is redistributed within the nucleobase molecules. For example, in the case of the cytosinium cation in a crystal, its electrostatic potential around the N7 amino group is more positive than around the other N—H (and C—H) groups, including even the N3 protonation site. For an isolated cation, the difference is much smaller, if any. In the CC crystal, chloride ions cluster around the N7 amino group (Fig. 2 ▸), thus cation–anion interactions may in addition be enhanced by charge-density redistribution in a cationic molecule. Moreover, the redistribution may reduce cation–cation electrostatic repulsions: in dimers AA1 and AA3 the regions of molecules with the least positive electrostatic potentials cluster together, and in the case of dimer AA2 the region of the molecule having the most positive electrostatic potential (C4—N7H_2_) stacks with the least positive one (C2=O2). Similar effects can be observed for the adeninium cation in ACH, *i.e.* the charges redistribute in such a way that regions of the nucleobase interacting directly with the chloride have the highest positive potential and that potential is more uniformly spread over N—H and C—H fragments. These latter observations remarkably resemble the results of natural bond analysis of intermolecular N—H⋯Cl and C—H⋯Cl interactions in nicotino­hydrazide dihydrochloride crystals (Kruszynski, 2011 ▸). Additionally, regions which are in direct contact with another cation have relatively less positive electrostatic potential (Figs. 3 ▸ and 7 ▸).

**Figure 8:**
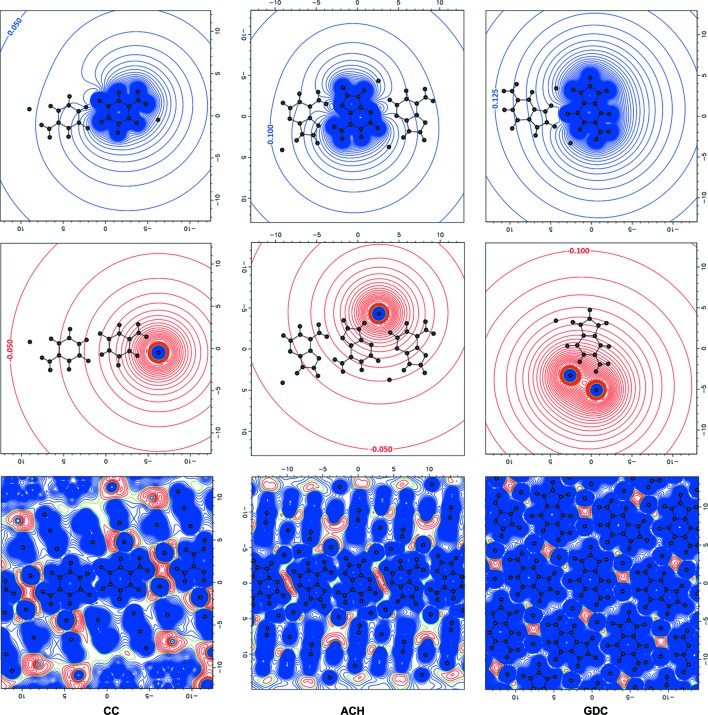
Electrostatic potentials (e Å^−1^) of (top) protonated nucleobase cations, (middle) chloride anions and (bottom) entire crystals from experimental charge densities in the plane of the nucleobase. Contour lines are drawn every 0.025 e Å^−1^. Blue denotes positive values, red negative and green zero.

**Figure 9:**
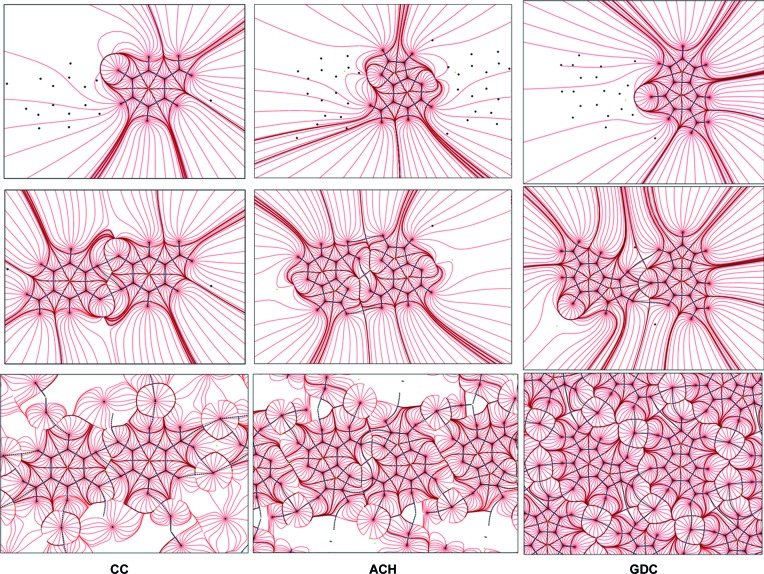
Gradient-field trajectory plots of the electrostatic potential of (top) nucleobases, (middle) nucleobase dimers AA1 and (bottom) entire crystals from experimental charge densities in the plane of the nucleobase.

It is also enlightening to analyse the electrostatic potential itself (Fig. 8 ▸) and the gradient field trajectories of the electrostatic potential (Fig. 9 ▸) of single chloride or nucleobase ions, dimers and entire crystals. From the plots of a single cytosinium cation and of the AA1 dimer, it is clearly visible that it is only after formation of the dimer that the electrostatic potential gradient lines in the plane of the nucleobase surround the dimer uniformly. Hence, the dimer electrostatic potential field outside of the dimer surface resembles more the field of an ideal cation (a cation with a uniformly distributed charge inside it). This may explain why this type of dimer is formed in nucleobase chlorides.

### 3.4. Interaction energies for dimers   

Following our analysis of the charge densities and electrostatic potentials of the studied molecules and crystals, we computed the energies of the intermolecular electrostatic interactions between whole molecules forming dimers as extracted from the crystal structures (Table 4 ▸). The list of analysed dimers includes not only dimers identified by atom–atom contacts being shorter than the sum of the van der Waals radii, but also from Hirshfeld surface analysis. In addition, interactions between the two closest chloride anions in the CC and ACH structures have been added to the analysis for the sake of comparison. To understand their role in intermolecular interactions in the studied dimers, we compared electrostatic energies with total interaction energies (*E*_tot_).

As one might expect for ionic structures, dimer interaction energies (*E*_tot_) are in general dominated by the net charges of the molecules, *i.e.* the energies are negative for dimers of molecules having opposite charges (*AB*-type dimers) and positive for dimers of molecules whose charges have the same sign (*AA*- or *BB*-type dimers). The absolute values of the energies are very high, much higher than those of neutral molecules (Jarzembska *et al.*, 2012 ▸; Jarzembska, Goral *et al.*, 2013 ▸; Jarzembska, Kamiński *et al.*, 2013 ▸), reaching values of around ±200 kcal mol^−1^ (1 kcal mol^−1^ = 4.184 kJ mol^−1^) for dimers in the GDC structure containing dication(s). Decomposition of the total interaction energies within the perturbation theory scheme (Table S3) confirms that it is indeed the electrostatic component (*E*_es_, the electrostatic interaction energy between two charge densities of isolated molecules) which contributes the most to the total energy, the other components being – with two exceptions (dimers AA1 in CC and ACH) – at least three times smaller in absolute value.

The *E*_es_ energies from the experimental crystal charge densities are systematically lower in absolute value than those from theoretical approaches relying on monomer and dimer gas-phase (not periodic) approximations (*UBDB*, DFT-SAPT). To understand the main source of this discrepancy, *E*_es_ energies can be contrasted with energies computed from Coulomb’s law applied to two point molecular charges placed at the distances of the centres of molecular mass; see *E*_Coul_ in Table 4 ▸. Although the point molecular charge is a very crude approximation of the true charge density, it adequately explains the overall trends observed for the *E*_es_ values in relation to the molecular charges and to distance. The *E*_Coul_ energies computed from experimental molecular charges are shifted towards smaller absolute values with respect to the *E*_Coul_ energies computed from formal charges. The shift is, in general, of the same magnitude as the shift in the *E*_es_ energies from experimental crystal charge densities with respect to the *E*_es_ energies from *UBDB* or DFT-SAPT. Thus, the fact that the molecules are not fully ionized in the crystal structures has a noticeable effect on the electrostatic energies computed for particular dimers.

A detailed analysis of why *E*_es_ deviates more from *E*_Coul_ for some dimers than for others, whether it can be explained by atom–atom interactions, and whether topological analysis of intermolecular bonding lines will tell more in that respect will be presented in our next paper.

A closer look at the aforementioned shifts identifies that there is a secondary effect influencing the *E*_es_ energies that is unrelated to differences in molecular charge. The effect can be attributed to a charge-density redistribution within the mol­ecules upon crystal formation, a redistribution already visible in the electrostatic potential at the molecular surfaces (see previous subsection). To quantify the effect we did the following simulation. We used the *UBDB* approach to build molecular electron densities of nucleobases rescaled to the molecular charges observed experimentally. In this way we obtained electron densities which have net charges as do those from the experiment, but they miss the effect of charge redistribution inside the molecule caused by the presence of neighbouring molecules. For the selected dimers we then computed the *E*_es_ energies from these charge densities (Table 4 ▸, values in parentheses in the *UBDB* column). The differences between these energies and those computed from the experimental charge densities quantify the effect of charge redistribution on the electrostatic interaction energies. The differences are small, up to around 5 kcal mol^−1^. Their values follow, to some extent, basic assumptions based on the electro­static potential at a molecular surface, especially in the case of CC. However, not all dimers gain from charge redistribution. In the case of GDC, the role of charge redistribution is unclear, both from the electrostatic potential and from the energetic point of view. It has to be noted, however, that the multipole model is generally able to describe electronic polarization qualitatively, but has some difficulties with a quantitative description of it (Bąk *et al.*, 2012 ▸).

The AA1 dimer in CC (and in ACH to some extent) deserves special attention. Despite the fact that it is a dimer of two cations, the negative electrostatic energy suggests that these two cations may form stable interactions. This is not only because the protonated nucleobases have charges lower than a formal +1 e in the crystalline state, but also because the charge redistributes within the cation. Another characteristic feature of the AA1 dimer in CC, and also in ACH, is the fact that the electrostatic interaction energies have the smallest absolute values when compared with the other dimers of AA type analysed here. In addition, the remaining components of the total interaction energy, like induction, exchange-repulsion and dispersion, contribute similar amounts of energy in absolute terms as the electrostatic component does, thus resembling the behaviour of dimers of neutral molecules (Czyżnikowska *et al.*, 2010 ▸). Apparently, protonated nucleobases in highly charged environments may form (meta)stable base-pair dimers and their interactions may not differ significantly from those among neutral (non-protonated) nucleobases. Similarity in interactions between neutral dimers and dimers of like-charged molecules is not a new concept and has already been reported in the literature for dimers of hydrogen oxalate (Macchi *et al.*, 2000 ▸), phosphates (Mata *et al.*, 2012 ▸, 2013 ▸), oxalic acids (Mata *et al.*, 2015 ▸) and carboxylic acids (Alkorta *et al.*, 2016 ▸), and for like-charged halogen-bonded complexes (Quiñonero *et al.*, 2016 ▸).

**Figure 10:**
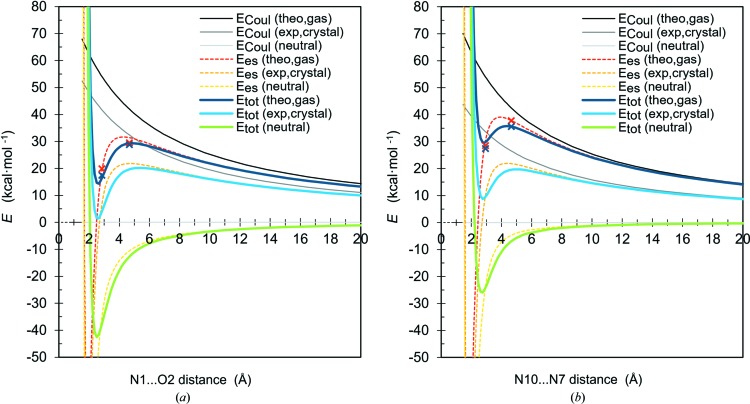
Total interaction energies *E*_tot_ (kcal mol^−1^), electrostatic contributions *E*_es_ (kcal mol^−1^) to total interaction energies, and electrostatic energies computed from point molecular charges *E*_Coul_ (kcal mol^−1^) for the AA1 dimers extracted from (*a*) the CC and (*b*) the ACH experimental crystal structures as a function of donor⋯acceptor distance (Å), simulating the dissociation curve along hydrogen bonding. *E*_tot_ (theo,gas) and *E*_es_ (theo,gas) are the energies according to the DFT-SAPT method applied to dimers of molecules bearing formal +1 e charge; *E*_tot_ (exp,crystal) and *E*_es_ (exp,crystal) are the energies from DFT-SAPT after rough correction taking into account the partial molecular charge observed from experiment; *E*_tot_ (neutral) and *E*_es_ (neutral) are the energies from DFT-SAPT after complete removal of electrostatic repulsion due to the presence of molecular charge; and *E*_Coul_ are the energies computed from various point molecular charges placed at the centres of molecular mass: +1 e (theo,gas), experimental charge (exp,crystal), 0 e (neutral). Dark-blue and red crosses denote *E*_tot_ (theo,gas) and *E*_es_ (theo,gas) DFT-SAPT energies, respectively, computed for geometries taken from dimer geometry optimization and transition state search in the gas phase.

To explore further the possibility of forming (meta)stable dimers with the AA1 dimers in CC and ACH, we have simulated interaction energy curves for the dissociating dimers as an isolated system in the gas phase using the DFT-SAPT approach (Fig. 10 ▸). The energies were computed for a series of dimers whose geometry was obtained by gradual extension or contraction of the hydrogen bond donor⋯acceptor distances, starting from the dimers’ geometry as observed in the crystals. The interaction energy curves show local minima and local maxima. Thus, the simulation confirms that the studied dimers are indeed capable of forming metastable dimers in the gas phase and the geometry of these dimers very much resembles the geometry present in the crystalline state. The energy barrier is high enough to suggest that these dimers might be kinetically stable and experimentally observable, even in isolation in the gas phase and most probably in solution. The existence of the true local minima and transition states, and the depths of the local energy wells, were further confirmed by geometry optimization and transition-state search in the gas phase using the DFT approach (Fig. 10 ▸). Analogous observations made for other kinds of same-charge dimers, not for nucleobases, are present in the literature (Gamrad *et al.*, 2015 ▸; Weinhold, 2017 ▸, 2018 ▸; Alkorta *et al.*, 2016 ▸; Grimme & Djukic, 2011 ▸; Shokri *et al.*, 2013 ▸; Mata *et al.*, 2015 ▸).

To understand the influence of charge transfer on the stability of the studied pairs, we have corrected the energies obtained for Coulombic repulsion by subtracting from them the difference between the *E*_Coul_ energy computed from the formal molecular charges and the *E*_Coul_ energy computed from the partial molecular charges found for the nucleobases in the crystals (Fig. 10 ▸). The charge transfer lowers the absolute values of the interaction energy at the minima and maxima and seems to increase the dissociation barriers. Moreover, the charge transfer as observed in crystals brings the energy of the local minimum very close to the negative values and very little is needed to achieve truly attractive interactions. The effect resembles, to some extent, the influence of solvent discussed in the literature cited above. However, to our knowledge, we are the first to show that intermolecular charge transfer is responsible for the conversion of metastable dimers of same-charge molecules into stable ones.

All the nucleobase dimers analysed here (which will be shown in our next paper), and the AA1 dimers in CC and ACH in particular, show that ionic molecules in ionic crystals are not only passive hostages of Coulombic compression (Braga *et al.*, 1998 ▸, 1999 ▸, 2002 ▸; D’Oria *et al.*, 2012 ▸), but they actively respond to it through intermolecular charge transfer, intramolecular electronic polarization and local attractive interactions like hydrogen bonding. The possibility of forming protonated nucleobase dimers in solution prior to crystallization should not be excluded.

### 3.5. Cohesive energies for crystals   

To sum up the analysis of the energies of intermolecular interactions in the crystals studied here, we computed the total crystal cohesive energies (*E*_coh_) as well as the electrostatic contributions to them (*E*_coh,es_) (Table 5 ▸). Crystal cohesive energies, analogous to dimers, result almost entirely from electrostatic interactions according to the *PIXEL* method. The other energy components, including polarization, are a couple of times smaller in their absolute values. As observed for the interaction energies for dimers, the electrostatic contribution seems to be a useful predictor for crystal cohesive energies in the ionic crystals included in this study. This could explain why the cohesive energy for GDC is much larger in absolute value than that of CC or ACH.

As expected for stable ionic crystals, the negative electrostatic energies between the cations and anions in the studied crystals dominate over the positive electrostatic energies among the cations and anions. Interestingly, a large portion of the electrostatic contribution to the cohesive energy in GDC can be explained simply by invoking the interactions of point molecular charges placed in the crystal at centres of molecular mass (see *E*_coh,es_ and *E*_coh,Coul_). In the case of CC, the shape of the cation and the charge distribution inside the molecule have almost as much influence on the overall electrostatic interactions in the crystal as molecular charges alone. For ACH, the molecular charges contribute even less than in the case of GDC and CC, because in this structure the charges are diluted by almost-neutral water molecules.

As in the case of electrostatic energies for dimers, the electrostatic contributions to the cohesive energies of the crystals are also very much influenced by the fact that the chlorides and nucleobases are not fully ionized (see values of *E*_coh,Coul_ for the experimental and *UBDB* models).

The electrostatic contributions to the crystal cohesive energies (*E*_coh,es_) computed from the experimental densities are very similar to these from the *UBDB* simulation, in which the molecular charge is the same as in the experiment but charge redistribution within the molecules is missing (Table 4 ▸, values in parentheses in the *UBDB* column). This suggests that electronic polarization, after intermolecular charge transfer is separated out, does not sum to large values when the whole crystal is analysed.

The electrostatic contributions to crystal cohesive energies (*E*_coh,es_) computed from multipolar models fitted to simulated theoretical structure factors are very similar to those from experiment, except for GDC. Most probably, the discrepancies in the level of charge transfer as established from these two approaches are the main source of disparity in the *E*_coh,es_ energies. It is uncertain which method (theory or experiment) is closer to the true value.

To sum up, two independent methods, *i.e.* experimental charge densities and periodic quantum mechanical calculations, gave values of crystal cohesive energies for the studied crystals at comparable levels. However, the relative difference of 5–20% might still not be very gratifying. There are many reasons for these discrepancies. Firstly, the *E*_coh_ energies from the periodic calculations were computed with reference to fully ionized molecules, which is not the case for *E*_coh,es_ from experiment. The energy of partial charge transfer from chloride to protonated nucleobase ions is missing from these calculations. Secondly, periodic calculations are likely to be affected by errors related to dispersion and/or correlation effects, whereas experimental results, apart from experimental errors, are limited by multipolar modelling. It is also uncertain how dispersion and exchange-repulsion effects present in the experimental crystal charge densities should be taken into account to compute their contribution to cohesive energies *via* summation over molecule–molecule interactions. Further studies are necessary to improve both methods and achieve better agreement.

## 4. Conclusions and outlook   

The present work contributes to our understanding of intermolecular interactions among protonated nucleobases and chloride counter-ions surrounding them in the solid state. For single crystals of cytosinium chloride (CC), adeninium chloride hemihydrate (ACH) and guaninium dichloride (GDC), we collected high-resolution X-ray diffraction data to determine crystal charge densities experimentally. On the basis of these, we have extensively analysed the molecular charges, electrostatic potentials and electrostatic interaction energies for molecular dimers and whole crystals. These experimental results were further supported by extensive theoretical calculations, including *UBDB* charge-density modelling, dimer interaction energy computations based on perturbation theory (*PIXEL*, DFT-SAPT) and periodic quantum mechanical computations (*CRYSTAL*) based on the variational principle.

As expected for ionic crystals, our results confirm that crystal cohesive energies and the interaction energies of ionic dimers identified in the crystalline state are dominated by contributions from electrostatic interactions. Electrostatic energies (*E*_es_) usually constitute 95% of the total interaction energy in these systems. However, the electrostatic inter­actions are not as strong as perturbation theory-based calculations suggest. This is because, according to the experimental data, the molecules included in this study are not fully ionized in the crystalline state. To our knowledge, this is the first experimental evidence, deeply explored in a quantitative manner from the point of view of interaction energy, that protonated nucleobases may bear a charge smaller than the formal one. Secondary effects of intramolecular charge redistribution, besides intermolecular charge transfer, are visible on molecular electrostatic potentials but seem to make minor contributions to the crystal cohesive energies.

Moreover, we show for the first time that singly protonated nucleobases are still capable of forming (meta)stable base pairs while surrounded by negative ions. This is because they interact strongly with each other through hydrogen bonding which overcomes cation–cation repulsion. The nature of this interaction does not differ much from those between neutral molecules. Chloride-to-nucleobase charge transfer and intramolecular charge-density polarization also contribute to this effect, which has not been observed experimentally before and which is discussed in detail in the context of same-charge dimers.

From a biological point of view, our finding is of great importance. Our study suggests that at least protonated adenine, as part of a nucleic acid, might be able to form stable homodimers of the type observed in the studied crystal, namely *trans* Hoogsteen/Hoogsteen edge base pairs. In the case of protonated cytosine, the particular base pair observed in the studied crystal cannot be formed by nucleic acids as it involves nitrogen atoms, which are bonded to a sugar moiety in nucleic acids. But it is still not ruled out that other types of base pair, including heterodimers, could be formed between two protonated bases and have metastable characteristics. Nucleic acids are highly charged polymers, bearing large negative charges due to their phosphate groups, often surrounded by many small cations and anions. Thus, they may impose a similar electric field on protonated bases as the chloride anions do in the studied crystals. What is important is that protonation of nucleobases is a well known mechanism by which these molecules change their properties towards specific recognition of particular partners. While analysing structures of nucleic acids and looking for the molecular basis of their specific interactions and functions, the possibility of attractive interactions between protonated nucleobases should not be excluded.

One may further speculate that base pairs which may exist in their protonated and neutral forms and which, while protonated, are metastable may have a unique ability to fine tune interactions between two nucleic acid chains. One can imagine that, in the case of the adenine–adenine *trans* Hoogsteen/Hoogsteen edge base pair (dimer AA1 in ACH), any changes in the hydrogen bonding at the N1 position will influence the partial charge of the base and thus regulate how stable the pair is. In addition, the N10⋯N7 distance might be very elongated before the pair will leave its metastable zone, even if stabilizing anions are removed from the close environment. Once the energy barrier of dissociation for the protonated pair has been overcome, a large amount of energy will be released (equivalent to the height of the maximum). This energy might be utilized for a conformational change of the interacting nucleic acid chains.

In fact, the adenine–adenine *trans* Hoogsteen/Hoogsteen edge base pair is the most common of all the types of adenine–adenine pairs present in the non-redundant set of RNA structures (http://ndbserver.rutgers.edu/ndbmodule/services/BPCatalog/bpCatalog.html): around 300 instances of this studied base pair were noted. For example, the studied pair is the only one present in the parallel double helix formed by two polyadenylic acid (poly A) chains (Gleghorn *et al.*, 2016 ▸; Ke *et al.*, 2009 ▸; Copp *et al.*, 2017 ▸; Petrovic & Polavarapu, 2005 ▸, 2006 ▸; Kim *et al.*, 2011 ▸; Safaee *et al.*, 2013 ▸). Moreover, the existence of the pair in its protonated form in the double helix, exactly as in the studied crystals, with phosphate groups in the position of chlorides and with a water molecule in the same place, is well proven in acidic media. However, there is quite a debate in the literature as to whether the pair is protonated or not at neutral pH. It is worth stressing that poly A tails are very common in some types of RNA. Also, the studied base pair is very common in crystal structures of small molecules deposited in the CSD. There are *ca* 70 such structures containing the pair, among which more than half are built from two adenines protonated at the N1 position or N9 derivatives of adenines. Currently, we are doing more comprehensive statistics on the homopairs of nucleobases and their derivatives in their neutral and protonated forms present in the CSD and we will publish the results soon. The results presented here explain why this particular base pair is observed so frequently.

The question of whether the observed protonated base pairs are structure-determining remains open. Further studies of the dimerization of like-charged nucleobases in solution are also necessary. Our experimental results show that the presence of appropriate external electric fields substantially influences how charged nucleobases interact with each other, thus allowing for attractive interactions between molecules of the same net charge. Most probably, the same mechanism may occur in states other than crystalline ones. Nucleobase pairing is definitely RNA/DNA structure-determining, and it would be interesting to investigate whether it still holds true for pairings of protonated nucleobases.

## Supplementary Material

Crystal structure: contains datablock(s) CC, ACH, GDC. DOI: 10.1107/S2052252518006346/lc5097sup1.cif

Structure factors: contains datablock(s) CC. DOI: 10.1107/S2052252518006346/lc5097CCsup2.hkl

Structure factors: contains datablock(s) ACH. DOI: 10.1107/S2052252518006346/lc5097ACHsup3.hkl

Structure factors: contains datablock(s) GDC. DOI: 10.1107/S2052252518006346/lc5097GDCsup4.hkl

Supporting information file. DOI: 10.1107/S2052252518006346/lc5097CCsup5.cml

Supporting information file. DOI: 10.1107/S2052252518006346/lc5097ACHsup6.cml

Supporting information file. DOI: 10.1107/S2052252518006346/lc5097GDCsup7.cml

Experimental details, and additional figures and tables. DOI: 10.1107/S2052252518006346/lc5097sup8.pdf

Raw diffraction frames URL: https://doi.org/10.18150/repod.7313736

Raw diffraction frames URL: https://doi.org/10.18150/repod.0481200

Raw diffraction frames URL: https://doi.org/10.18150/repod.8020589

CCDC references: 1539172, 1539174, 1539173

## Figures and Tables

**Table:** Values in parentheses are for the highest resolution shell.

	Cytosinium chloride (CC)	Adeninium chloride hemihydrate (ACH)	Guaninium dichloride (GDC)
Empirical formula	C_4_H_6_N_3_O·Cl	C_5_H_6_N_5_·Cl·0.5(H_2_O)	0.5(C_5_H_7_N_5_O)·Cl
*M*_r_ (g mol^−1^)	147.56	180.60	112.25
Temperature (K)	89.9 (3)	89.9 (3)	100
Crystal system, space group	Monoclinic, *P*2_1_/*n*	Monoclinic, *P*2/*n*	Orthorhombic, *Pnma*
*a*, *b*, *c* (Å)	8.1481 (1)	8.6936 (1)	13.5939 (5)
	6.8774 (1)	4.8189 (1)	6.4841 (2)
	10.9947 (1)	17.6971 (11)	9.8600 (4)
α, β, γ (°)	90	90	90
	95.967 (1)	93.526 (1)	90
	90	90	90
Volume (Å^3^)	612.78 (1)	739.99 (1)	869.10 (5)
*Z*	4	4	8
*D*_*x*_ (Mg m^−3^)	1.600	1.621	1.712
μ (mm^−1^)	0.535	0.462	0.716
Crystal colour and shape	Transparent colourless block	Transparent colourless block	Opaque colourless block
Crystal size (mm)	0.16 × 0.12 × 0.05	0.34 × 0.20 × 0.07	0.16 × 0.16 × 0.06
Diffractometer	Agilent Supernova	Agilent Supernova	Bruker APEXII ULTRA
Radiation type	Mo *K*α	Mo *K*α	Mo *K*α
*F*(000)	304	372	456
Limiting indices	−16 → *h* → 16	−22 → *h* → 22	−30 → *h* → 30
	−13 → *k* → 13	−12 → *k* → 12	−14 → *k* → 14
	−22 → *l* → 22	−45 → *l* → 45	−22 → *l* → 22
Collected reflections	82389	101278	232874
Independent reflections	5144	13139	5605
Resolution (sinθ/λ) (Å^−1^)	0.073–1.002 (0.967–1.002)	0.057–1.285 (1.241–1.285)	0.063–1.138 (1.098–1.138)
Average redundancy	16 (10.6)	7.7 (4.5)	41.5 (14.3)
Mean *I*/σ(*I*)	21.6 (10.0)	17.3 (7.0)	20.2 (4.6)
Completeness (%)	100 (100)	99.6 (95.6)	99.5 (98.5)
Merging *R* factors (%)	*R*_1_ = 4.23 (15.78)	*R*_1_ = 3.58 (14.54)	*R*_1_ = 5.86 (40.52)
	*R*_2_ = 3.53 (13.01)	*R*_2_ = 2.28 (11.41)	*R*_2_ = 3.70 (31.13)
	*R*_w_ = 6.71 (19.09)	*R*_w_ = 6.74 (16.47)	*R*_w_ = 5.76 (35.63)
	*R*_m_ = 1.00 (4.32)	*R*_m_ = 1.24 (6.68)	*R*_m_ = 0.88 (10.66)
Absorption correction	Multi-scan	Multi-scan	Multi-scan

**Table:** 

Multipole refinement statistics for ionic Cl^−1^ scattering radial functions (top row of each entry) and for neutral Cl^0^ (bottom row of each entry, in italics)			
*I*/σ(*I*) cutoff	2.0	2.0	2.0
No. of observed reflections	4590	11241	4482
No. of restraints	6	7	7
No. of parameters	276	358	322
*R*(*F*), *wR*_2_(*F*) (%)	1.59, 2.37	2.17, 2.83	1.79, 1.90
	*1.58*, *2.37*	*2.19*, *2.84*	*1.77*, *1.89*
*R*(*I*), *wR*_2_(*I*) (%)	1.85, 4.28	2.08, 5.03	1.84, 3.58
	*1.74*, *4.28*	*2.07*, *5.04*	*1.68*, *3.56*
*w*GOF on *F* ^2^	1.21	1.21	1.05
	*1.22*	*1.21*	*1.04*
Largest difference peak/hole (e Å^−3^)	0.27, −0.26	0.29, −0.24	0.44, −0.32
	*0.26*, *−0.26*	*0.30*, *−0.24*	*0.43*, *−0.33*
Rigid-bond r.m.s. (Å^2^ × 10^−4^)	2.2	1.6	2.5
	*2.0*	*1.6*	*2.6*

**Table:** 

	Cytosinium chloride (CC)	Adeninium chloride hemihydrate (ACH)	Guaninium dichloride (GDC)
Independent reflections	11286	13625	8354
Resolution range (sinθ/λ) (Å^−1^)	0.07–1.30	0.06–1.30	0.06–1.30
No. of observed reflections	11286	13625	8354
No. of restraints	1	1	1
No. of parameters	172	222	229

**Table:** 

Theoretical structure factors computed for experimental geometry with the use of ionic Cl^−1^ scattering radial functions (top row of each entry) and neutral Cl^0^ ones (bottom row of each entry, in italics)			
*R*(*F*) (%)	0.65	0.69	0.62
	*0.75*	*0.78*	*0.73*
*R*(*I*) (%)	1.01	1.07	0.94
	*1.21*	*1.24*	*1.14*
Largest difference peak/hole (e Å^−3^)	0.28, −1.14	0.29, −1.11	0.31, −1.13
	*0.28*, *−1.76*	*0.29*, *−1.74*	*0.30*, *−1.75*

**Table:** 

Theoretical structure factors computed for optimized geometry with the use of ionic Cl^−1^ scattering radial functions (top row of each entry) and neutral Cl^0^ ones (bottom row of each entry, in italics)			
*R*(*F*) (%)	0.65	0.70	0.62
	*0.75*	*0.78*	*0.73*
*R*(*I*) (%)	1.02	1.08	0.96
	*1.22*	*1.24*	*1.14*
Largest difference peak/hole (e Å^−3^)	0.30, −1.13	0.30, −1.10	0.31, −176
	*0.31*, *−1.75*	*0.30*, *−1.72*	*0.24*, *−1.14*

**Table 3:** Intermolecular contacts in the CC, ACH and GDC structures that are shorter than the sum of the van der Waals radii (Bondi, 1964 ▸), as established from experiment (upper values in each entry) and from periodic optimization of crystal geometry (lower values in each entry, in italics) For the symmetry operations required to build particular dimers, see Table S2 in the supporting information. In the ‘Interaction type’ column, HB denotes a hydrogen bond.

Dimer	Interaction	Interaction type	*D*⋯*A* (Å)	*D*—H† (Å)	H⋯*A* (Å)	*D*—H⋯*A* † (°)
Cytosinium chloride (CC)						
AA1	N1—H1⋯O2	HB	2.7782 (5)	1.01 (4)	1.77 (4)	175.61 (14)
			*2.7671*	*1.038*	*1.734*	*173.0*
AA2	C2⋯C4	π⋯π	3.3118 (4)			
			*3.347*			
AA3	C6—H6⋯O2	HB	3.1093 (6)	1.06 (3)	2.33 (2)	129.(1)
			*3.0957*	*1.087*	*2.292*	*129.2*
AB1	N7—H7*B*⋯Cl1	HB	3.3042 (4)	1.016 (5)	2.362 (7)	153.8 (4)
			*3.2673*	*1.027*	*2.300*	*156.6*
	C5—H5⋯Cl1	HB	3.4600 (4)	1.084 (5)	2.545 (8)	141.5 (4)
			*3.4810*	*1.087*	*2.580*	*139.7*
AB2	N7—H7*B*⋯Cl1	HB	3.3079 (5)	1.016 (5)	2.865 (10)	107.0 (6)
			*3.3289*	*1.027*	*2.962*	*101.9*
	*N7-H7*A*⋯Cl1*	*HB*				
			*3.3289*	*1.019*	*2.933*	*103.9*
AB3	C2⋯Cl1	π⋯Cl^−^	3.3399 (3)			
			*3.337*			
AB4	N3—H3⋯Cl1	HB	3.0232 (4)	1.015 (5)	2.022 (6)	168.6 (3)
			*3.0286*	*1.051*	*1.994*	*167.6*
	N7—H7*A*⋯Cl1	HB	3.4577 (4)	1.015 (5)	2.619 (8)	140.0 (4)
			*3.4996*	*1.019*	*2.686*	*136.9*
Adeninium chloride hemihydrate (ACH)						
AA1	N10—H10*A*⋯N7	HB	2.8980 (3)	1.009 (3)	1.937 (6)	158.2 (5)
			*2.8728*	*1.035*	*1.874*	*161.0*
	*N10-H10*A*⋯C8*	*HB*				
			*3.4843*	*1.035*	*2.684*	*134.0*
AA2	C2—H2⋯N3	HB	3.2888 (3)	1.083 (3)	2.734 (14)	111.5 (5)
			*3.324*	*1.092*	*2.737*	*113.3*
AA3	C2⋯C4	π⋯π	3.3430 (3)			
			*3.365*			
AB1	N10—H10*B*⋯Cl1	HB	3.17604 (18)	1.009 (3)	2.255 (8)	151.2 (7)
			*3.1679*	*1.028*	*2.214*	*153.5*
AB2	C8—H8⋯Cl1	HB	3.6059 (2)	1.083 (3)	2.605 (7)	153.1 (5)
			*3.5587*	*1.090*	*2.548*	*153.8*
AB3	N9—H9⋯Cl1	HB	3.0978 (2)	1.009 (3)	2.109 (4)	165.9 (3)
			*3.1187*	*1.037*	*2.099*	*167.4*
AB4	C2—H2⋯Cl1	HB	3.7134 (2)	1.083 (3)	2.699 (7)	155.6 (5)
			*3.679*	*1.092*	*2.676*	*152.3*
AW1	N1—H1⋯O1	HB	2.8251 (2)	1.009 (3)	1.834 (4)	166.4 (4)
			*2.7952*	*1.045*	*1.762*	*169.3*
BW1	O1—H1*A*⋯Cl1	HB	3.0742 (3)	0.967 (3)	2.118 (3)	169.63 (18)
			*3.0727*	*0.992*	*2.090*	*170.5*
Guaninium dichloride (GDC)						
AA1	C8—H8⋯O10	HB	2.7809 (6)	1.0830 (11)	2.245 (9)	108.2 (5)
			*2.7579*	*1.086*	*2.203*	*109.2*
	N9—H9⋯O10	HB	2.7591 (7)	1.0150 (11)	2.260 (12)	108.7 (7)
			*2.7475*	*1.037*	*2.214*	*110.0*
AB1	N11—H11B⋯Cl1	HB	3.0600 (3)	1.0141 (11)	2.084 (3)	160.8 (3)
			*3.0444*	*1.032*	*2.066*	*157.2*
	N3—H3⋯Cl1	HB	3.1960(4)	1.0140 (11)	2.274 (5)	150.6 (4)
			*3.2195*	*1.034*	*2.320*	*144.7*
AB2	N1—H1⋯Cl1	HB	3.0467 (4)	1.0140 (11)	2.0357 (15)	174.57 (11)
			*3.0509*	*1.048*	*2.007*	*174.3*
AB3	C8—H8⋯Cl1	HB	3.8684 (4)	1.0830 (11)	2.826 (3)	161.4 (4)
			*3.793*	*1.085*	*2.762*	*158.5*
AB4	N9—H9⋯Cl2	HB	3.1345 (4)	1.0150 (11)	2.204 (6)	151.7 (6)
			*3.1424*	*1.037*	*2.205*	*149.5*
AB5	N7—H7⋯Cl2	HB	3.0733 (4)	1.0151 (11)	2.076 (2)	167.0 (2)
			*3.0742*	*1.050*	*2.043*	*166.8*
AB6	N11—H11*A*⋯Cl2	HB	3.1841 (4)	1.0140 (11)	2.271 (5)	149.1 (3)
			*3.1371*	*1.029*	*2.213*	*148.5*
AB7	C8⋯Cl1	π⋯Cl^−^	3.2546 (1)			
			*3.265*			
AB8	C2⋯Cl2	π⋯Cl^−^	3.4244 (1)			
			*3.431*			

† Note that standard uncertainties for parameters involving hydrogen atoms are affected by the use of *X*—H restraints.

**Table 4:** Total interaction energies *E*_tot_ (kcal mol^−1^) according to the SAPT method and electrostatic contributions (kcal mol^−1^) to the total interaction energy for selected dimers extracted from the CC, ACH and GDC crystal structures according to different methods *E*_es_ is the electrostatic energy computed from exact integration of charge densities and *E*_Coul_ is the electrostatic energy computed from the monopole moments of the molecules. For the symmetry operations required to build particular dimers, see Table S2 in the supporting information.

			DFT-SAPT		*UBDB*		Experimental	
Dimer	No. of hydrogen bonds	Centre of mass distance (Å)	*E*_tot_	*E*_es_	*E*_es_ †	*E*_Coul_	*E*_es_ ‡	*E*_Coul_
Cytosinium chloride (CC)								
AA1	2	6.06	20.0	16.6	14.0 (3.6)	54.8	−0.8 (−6.7)	42.3
AA2	0	3.46	55.4	61.9	60.8 (45.4)	96.1	42.8 (38.9)	74.1
AA3	1	6.74	38.1	39.8	40.0 (29.6)	49.3	27.8 (25.6)	38.0
AA4	0	5.38	64.3	68.5	68.5 (53.4)	61.6	57.7 (55.4)	47.5
AA5	0	6.88	46.2	50.0	50.3 (38.9)	48.3	38.9 (37.4)	37.2
AA6	0	6.74	52.2	56.2	57.6 (44.9)	49.3	42.7 (44.3)	38.0
AB1	2	4.84	−101.1	−96.8	−95.2 (−76.1)	−68.6	−80.2 (−78.7)	−52.9
AB2	1	5.83	−84.5	−79.7	−76.6 (−60.9)	−57.0	−65.3 (−61.4)	−43.9
AB3	0	3.31	−85.3	−82.8	−79.1 (−61.0)	−100.4	−60.1 (−57.2)	−77.4
AB4	2	3.96	−108.9	−106.2	−107.7 (−87.2)	−83.8	−86.7 (−85.0)	−64.6
AB5	0	4.89	−74.9	−69.7	−70.0 (−54.2)	−67.9	−52.1 (−51.7)	−52.4
AB6	0	4.51	−75.3	−70.2	−70.4 (−54.4)	−73.6	−52.8 (−51.3)	−56.7
BB1	0	3.99	75.4	80.9	83.1 (64.4)	83.2	63.8 (59.8)	64.2
Adeninium chloride hemihydrate (ACH)								
AA1	2	6.08	33.2	29.5	37.0 (16.2)	54.6	14.4 (8.0)	34.4
AA2	2	6.91	40.1	41.4	40.3 (23.9)	48.1	24.5 (27.9)	30.3
AA3	0	4.60	50.7	55.2	52.6 (31.2)	72.3	33.3 (35.1)	45.5
AA4	0	4.82	51.4	55.7	55.8 (34.4)	68.9	35.1 (35.6)	43.4
AA5	0	4.13	51.0	56.3	57.6 (33.3)	80.3	32.8 (32.7)	50.6
AB1	1	5.67	−91.2	−86.2	−88.1 (−62.9)	−58.6	−63.1 (−64.2)	−38.7
AB2	1	5.93	−65.9	−61.0	−58.7 (−38.4)	−56.0	−45.3 (−42.0)	−37.0
AB3	1	4.92	−84.7	−81.1	−82.5 (−56.5)	−67.5	−54.3 (−61.8)	−44.6
AB4	1	5.75	−78.8	−72.7	−69.0 (−46.6)	−57.8	−49.8 (−53.6)	−38.1
AB5	0	4.79	−82.4	−76.7	−76.5 (−51.8)	−69.4	−50.7 (−52.9)	−45.8
AB6	0	5.56	−62.3	−57.7	−57.5 (−37.1)	−59.7	−40.4 (−40.2)	−39.4
AB7	0	5.33	−63.7	−59.4	−60.5 (−39.3)	−62.3	−42.2 (−42.5)	−41.2
BB1	0	4.26	70.4	76.6	78.0 (53.7)	77.9	54.2 (59.0)	53.9
AW1	1	4.75	−11.0	−14.8	−16.9 (−9.0)	0.0	−7.7 (−6.1)	4.2
BW1	1	3.03	−14.3	−18.5	−19.1 (−24.1)	0.0	−23.8 (−30.3)	−6.9
BW2	0	3.66	2.4	5.7	8.1 (1.0)	0.0	0.6 (−2.4)	−5.7
Guaninium dichloride (GDC)								
AA1	2	7.64	154.0	162.1	159.0 (100.1)	173.9	101.2 (110.5)	115.0
AA2	0	6.31	180.7	192.5	187.3 (121.5)	210.5	117.3 (136.0)	139.3
AA3	0	5.78	223.2	239.2	245.0 (165.2)	229.8	165.4 (189.4)	152.0
AB1	2	4.78	−191.8	−181.4	−184.3 (−126.1)	−139.0	−123.1 (−143.6)	−88.2
AB2	1	4.73	−164.0	−153.6	−153.3 (−101.2)	−140.4	−98.5 (−115.4)	−89.0
AB3	1	6.58	−128.9	−119.5	−119.0 (−75.6)	−100.9	−80.3 (−91.0)	−64.0
AB4	1	4.70	−188.4	−169.9	−177.9 (−127.0)	−141.2	−123.0 (−137.6)	−97.3
AB5	1	4.71	−162.8	−151.5	−144.6 (−99.9)	−141.0	−95.4 (−108.8)	−97.1
AB6	1	6.06	−148.6	−138.5	−136.3 (−99.0)	−109.6	−95.3 (−106.6)	−75.5
AB7	0	4.44	−151.8	−144.2	−144.9 (−99.1)	−149.7	−91.5 (−107.4)	−94.9
AB8	0	3.31	−162.5	−156.0	−151.5 (−104.3)	−199.7	−102.1 (−116.7)	−137.5
BB1	0	3.69	80.1	85.6	89.6 (59.3)	89.9	57.9 (65.2)	59.4

† Values in parentheses in this column are energies computed from the *UBDB* model scaled to the values of molecular charges observed experimentally.

‡ Values in parentheses in this column are energies computed from the multipole model fitted to theoretical structure factors computed from the optimized crystal structure.

**Table 5:** Cohesive energies (*E*_coh_; kcal mol^−1^) and electrostatic contributions to the cohesive energies (*E*_coh,es_, *E*_coh,Coul_; kcal mol^−1^) for the CC, ACH and GDC crystals computed from different methods for experimental geometries and for optimized theoretical geometries (values in italics) Energies are given per ionic pair (including halves of water molecules in the case of ACH). ND denotes values that were not determined due to limitations of the method.

*CRYSTAL*		*PIXEL*		*UBDB*		Experimental	
*E*_coh_	*E*_coh,es_	*E*_coh_	*E*_coh,es_	*E*_coh,es_ †	*E*_coh,Coul_	*E*_coh,es_	*E*_coh,Coul_
Cytosinium chloride (CC)							
−163.6	−152.4	−179.8‡	−173.9	−183.0	−123.5	−154.2	−80.6
−*164.5*	−*155.2*			(−152.1)			
Adeninium chloride hemihydrate (ACH)							
−173.6	−153.3	ND	ND	−188.9	−69.0	−148.3	−45.4
*−173.3*	*−160.6*			(−144.6)			
Guaninium dichloride (GDC)							
−395.63	−351.6	ND	ND	−449.1	−397.3	−309.1	−262.6
*−435.21*	*−363.3*			(−319.9)			

† Values in brackets in this column are energies computed from the *UBDB* model scaled to the values of molecular charges observed experimentally.

‡ The remaining contributions to *E*_coh_ are: *E*_coh,pol_ (*PIXEL*) = −26.0 kcal mol^−1^, *E*_coh,disp_ (*PIXEL*) = −35.0 kcal mol^−1^ and *E*_coh,rep_ (*PIXEL*) = 55.0 kcal mol^−1^.
