# Supplementary material for: Protonated nucleobases are not fully ionized in their chloride salt crystals and form metastable base pairs further stabilized by the surrounding anions
Source: IUCrJ. 2018 Jun 8;5(Pt 4):449–69. doi: 10.1107/S2052252518006346 (PMC6038959; doi:10.1107/S2052252518006346)
Supplement: Supplementary file 8 [file m-05-00449-sup8.pdf]

IUCrJ

Volume 5 (2018)

Supporting information for article:

Protonated nucleobases are not fully ionized in their chloride salt crystals and form metastable base pairs further stabilized by the surrounding anions

Prashant Kumar, Malgorzata Katarzyna Cabaj, Aleksandra Pazio and Paulina Maria Dominiak

Supporting Information

Protonated nucleobases are not fully ionized in their chloride salt crystals and form metastable base pairs further stabilized by the surrounding anions

Prashant Kumar, Małgorzata Katarzyna Cabaj, Aleksandra Pazio and Paulina Maria Dominiak*

Biological and Chemical Research Center, Department of Chemistry, University of Warsaw,

ul. Żwirki i Wigury 101, 02-089 Warszawa, Poland. E-mail: pdomin@chem.uw.edu.pl

2. Experimental and computational methods – detailed description

2.1. Experimental charge density models

Crystallization, data collection and processing. In order to obtain charge density quality, single crystals, adenine ($3.7 \cdot 10^{-3}$ mol) or guanine ($3.3 \cdot 10^{-3}$ mol) in their neutral form were dissolved in a mixture of 5.0 ml distilled water and few drops of 38% hydrochloric acid solution. The obtained suspensions were subsequently heated to 80 °C and heating was continued until the substrates dissolved entirely. The solutions were left in vials covered with paraffin film at 37 °C. Prismatic crystals of good quality were obtained after one month. As for cytosinium chloride, equimolar amounts of cytosine and 4-thiouracil ($1.5 \cdot 10^{-4}$ mol) were dissolved in 5.0 ml of distilled water and a few drops of 1M HCl. The mixture was stirred in ultrasound bath and heated to approximately 50 °C, until both compounds completely dissolved. Solutions were left for evaporation at room temperature in cytosinium chloride (CC) case, and in 37 °C in adeninium chloride hemihydrate (ACH) and guaninium dichloride (GDC) case. Good quality crystals were obtained after one month. All aforementioned compounds were purchased from the Sigma-Aldrich Corporation.

For cytosinium chloride (CC) and adeninium chloride hemihydrate (ACH) crystal samples, subatomic resolution X-ray measurements were performed at 90 K on an Agilent Technologies SuperNova four-circle diffractometer equipped with a micro-focus sealed tube and Eos CCD detector. The temperature was controlled with an Oxford Cryosystems low-temperature nitrogen gas-flow device (Cryostream Plus). The crystals were mounted on a goniometer head using nylon loop and placed 60 mm from the detector. For CC a total of 3836 frames were collected in 39 runs using ω scan; rotation width of 1.0° and exposure time in the range of 4 - 10 seconds. Similarly, for ACH a total of 3195 frames were collected in 67 runs using ω scan, a rotation width of 1.0° and an exposure time in the range of 12.50 - 110 seconds. For guaninium dichloride (GDC) crystals, high-resolution single crystal X-ray measurement was carried out on a Bruker APEX II ULTRA single-crystal diffractometer with a TXS rotating anode (Mo Kα, radiation λ = 0.71073 Å) equipped with a CCD-type area detector, multilayer optics and an Oxford Cryostream low temperature attachment set to 100 K. A transparent cube-shaped crystal sample of GDC was attached to a cryogenic nylon loop, mounted on a goniometer head and positioned 50 mm from the detector. A total of 12412 frames were collected in 44 runs to obtain the high redundancy data. Diffraction data was collected using the ω and φ scan method with a rotation width of 0.5° and the exposure time in the range of 20 - 60 seconds.

For CC and ACH, the determination of unit cell parameters, integration of reflection intensities, and data reduction, including multiscan absorption corrections, were performed using CrysAlis PRO Version 1.171.36.32 (CrysAlis, 2013). Finally, reflection merging was carried out with the SORTAV program (Blessing, 1987; Blessing, 1989; Blessing, 1995; Blessing, 1997). Whereas for GDC, the determination of unit cell parameters, integration of the reflection intensities, and data reduction were performed with the APEX2 suite of programs (integration was carried out with SAINT V8.27B) (Bruker, 2013) and the multiscan absorption correction, scaling and merging of reflection data were carried out with the SORTAV program (Blessing, 1987; Blessing, 1989; Blessing, 1995; Blessing, 1997).

Structure solution and refinement. Using Olex2 (Dolomanov *et al.*, 2009), the structures were solved with the ShelXS (Sheldrick, 2008) program with direct methods and refined with the olex2.refine refinement package with

Gauss-Newton minimization using the independent atom model (IAM).

Multipole refinements were performed in the MoPro Suite software package (Guillot *et al.*, 2001; Jelsch *et al.*, 2005) with the use of the Stewart-Hansen-Coppens multipolar model (Stewart *et al.*, 1975; Hansen & Coppens, 1978). In the multipole density formalism, the molecular electron density is expanded in pseudoatom density contributions. The density of each pseudoatom is given by:

$$\rho_i(r) = \rho_{core}(r) + P_{val}\kappa^3\rho_{val}(\kappa r) + \sum_{l=1}^{l_{max}} \kappa'^3 R_l(\kappa'\zeta r) \sum_{m=-l}^{m=l} P_{lm} d_{lm}(\theta,\phi) \quad (1)$$

where ρ_{core} and ρ_{val} are spherical and valence densities, respectively. The third term contains the sum of angular function $d_{lm}(\theta,\phi)$ to account for aspherical deformations. The coefficients P_{val} and P_{lm} are multipole populations for the spherical and multipole density, respectively. The κ and κ' are the scaling parameters which determine the expansion/contraction of spherical and multipolar valence densities, respectively.

For each sample, refinement was performed against structure factor amplitudes (F) and only those reflections fulfilling the $I \geq 2\sigma(I)$ conditions were taken into account as this was found to produce the best results. The initial atomic coordinates, x, y and z, for all atoms, anisotropic atomic displacement parameters (U_{ij}'s) for non-hydrogen atoms and isotropic atomic displacement parameters for hydrogen atoms were taken from the IAM refinement. With the use of the LSDB (Volkov, Li *et al.*, 2004) program, all deformation parameters were defined with respect to their local Cartesian coordinate systems and the initial multipolar and contraction-expansion parameters for nucleobases and water molecules were transferred from UBDB2011 (Jarzembska & Dominiak, 2012). Multipole expansion was truncated at the hexadecapole ($l_{max} = 4$) and quadrupole ($l_{max} = 2$) levels for all non-hydrogen and hydrogen atoms respectively. For hydrogen atoms, only the bond-directed dipoles and quadrupole populations (i.e. Dz and Qz^2) were refined. Special positional constraints for occupancy, coordinates, and thermal and multipolar parameters were applied to all atoms in GDC and to the O1 atom in ACH. For each sample P_{lm} parameters for all atoms were constrained to the local symmetry suggested by the LSDB program. Each atom was assigned with core and spherical-valence scattering factors derived from Su and Coppens atomic wave functions for neutral atom configurations except chlorine atoms (Su & Coppens, 1998). In the case of chlorine atoms two possibilities were investigated: the Cl^{-1} ion scattering radial function and Cl^{-1} ion electronic configurations (core: $1s^22s^22p^6$, valence: $3s^23p^6$), or the Cl^0 neutral scattering radial function and Cl^0 neutral electronic configurations (core: $1s^22s^22p^6$, valence: $3s^23p^5$). The anomalous dispersion coefficients were taken from Kissel *et al.* (Kissel *et al.*, 1995). The contraction-expansion parameters, κ and κ' for all hydrogen atoms were kept fixed at the UBDB2011 values during refinements. To eliminate instabilities in the refinements, it was necessary to constrain κ' parameters of the deformation functions to 1.0 for chlorine atoms. The values of the U_{ij} parameters for hydrogen atoms were estimated from the SHADE 3.0 server (Madsen, 2006); the values were not refined but updated in-between refinement stages until convergence. The X-H distances were restrained to the averaged values obtained from neutron diffraction studies (Allen & Bruno, 2010) with a restraint σ of 0.004 Å. Additionally it was found that the Cl1 atom of ACH undergoes noticeable anharmonic motion. Gram-Charlier (GC) coefficients (Kuhs, 1983; Johnson, 1969; Scheringer, 1985) for up to the third order were used to successfully model anharmonic motion, whereas the physical reliability of the anharmonic model was confirmed by the probability density function (PDF) computed with the MolIso program (Hubschle & Luger, 2009). It is probable

that also in the case of CC and GDC, anharmonic motion of Cl atoms is present, but the resolution of the data does not allow for reliable refinement of GC coefficients.

The detailed general refinement procedure involved the following refinement steps:

(i) scale factor refinement (which was also refined in almost all other stages);

(ii) high-order refinement ($\sin\theta/\lambda$ > 0.7 Å^{-1}) of the x, y, z and U_{ij} parameters was carried out for non-hydrogen atoms. Then, considering the whole resolution range, refinement of the scale factor, x, y, z, and U_{iso} parameters of the H atoms was carried out. These steps were repeated until convergence was obtained;

(iii) the anisotropic ADPs (U_{ij}) of the H atoms were estimated using the SHADE program and were kept constrained to the SHADE values in subsequent refinements;

(iv) multipole population parameters $P_{val}$and P'_{lm} in a stepwise manner;

(v) all multipole population parameters and structural parameters simultaneously followed by re-estimation of the U_{ij} from SHADE;

(vi) for CC and GDC block refinement of: non-hydrogen atom κ parameters (first block), step no.(v) (second block) and non-hydrogen atom κ' parameters (third block); for ACH block refinement of: non-hydrogen atom κ parameters (first block), step #(v) (second block), non-hydrogen atom κ' parameters (third block) and the third-order anharmonic parameters for the Cl atom [no scale factor refined; $\sin\theta/\lambda$ > 1.15 Å^{-1}] (fourth block).

(vii) all multipole population parameters, non-hydrogen atom κ parameters and structural parameters simultaneously.

The outcomes of multipole refinements were verified by examining R-factors, GOFs and residual densities, Table 1. The evaluation was additionally supported by the *JNK2RDA* program (Meindl & Henn, 2008) and the *XDRKplot* program implemented in the *WinGX* program package (Farrugia, 2012), see Figures S1– S4. An ORTEP diagram (Spek, 2009) with labelling of atoms is shown in the Figure 1. The CIF files can be retrieved from the Cambridge Structural Database (CSD) (Groom *et al.*, 2016) (deposition numbers: CCDC 1539172-1539174). Sets of raw diffraction frames and the associated data are accessible online under the following DOIs: 10.18150/repod.7313736, 10.18150/repod.0481200 and 10.18150/repod.8020589 (Repository for Open Data, Interdisciplinary Centre for Mathematical and Computational Modelling, University of Warsaw, Warsaw, Poland).

2.2. UBDB charge density models

UBDB models of charge densities for CC, ACH and GDC crystals were built on experimental geometries using LSDB (Volkov, Li *et al.*, 2004) and the UBDB2011 (Jarzembska & Dominiak, 2012) version of databank. Chlorine atoms were treated as spherical anions with Cl^{-1} ion scattering radial function and Cl^{-1} ion electronic configurations (core: $1s^22s^22p^6$, valence: $3s^23p^6$). Core and spherical-valence factors for each atom were taken from Su and Coppens' atomic wave functions (Su & Coppens, 1998). For the majority of calculations, the net charge of each molecule was scaled separately to its formal value: +1e for cytosinium and adeninium, +2 e for guaninium, 0 e for water molecules and -1 e for chloride anions. For alternative simulations (as explained further in the text), net molecular charges were scaled to experimentally observed values.

2.3. Periodic quantum mechanical calculations

Periodic quantum mechanical calculations based on the variational principle were applied to compute theoretical charge densities, theoretical structure factors and cohesive energies (E_{coh}). E_{coh} were computed using (Civalleri *et al.*, 2008):

$$E_{coh} = \frac{1}{Z} E_{bulk} - E_{mol} \quad (2)$$

where $E_{bulk}$is the total energy of a system (per unit cell) and E_{mol} is the energy of a molecule extracted from the bulk. Z stands for the number of molecules (here ionic pairs, including half of water molecule in the case of ACH) in the unit cell. The Crystal14 package (Dovesi *et al.*, 2014a; Dovesi *et al.*, 2014b) was used at the DFT-B3LYP/pVDZ level of theory (Becke, 1988; Perdew, 1986; Lee *et al.*, 1988; Dunning, 1989). The computations were done in two versions: for experimental and optimized geometry. During optimization, cell parameters were fixed while atom positions were allowed to vary. The Grimme dispersion correction (Grimme, 2006) was applied for all calculations whereas correction for basis set superposition error (BSSE) (Civalleri *et al.*, 2008) was applied for the computation of cohesive energy following the supermolecular approach (Maschio *et al.*, 2011). To determine the BSSE, subsystem calculations were performed using ghost atoms selected up to 5 Å distant from the considered molecule in a crystal lattice. The level of accuracy in evaluating the Coulomb and exchange series was controlled by five thresholds, for which values of 10^{-7} , 10^{-7}, 10^{-7}, 10^{-7} , and 10^{-25} were used. The irreducible Brillouin zone was sampled using 170 *k*-points (the shrinking factor of the reciprocal space net was set to 8). See Table S1 in SI for further details.

Static theoretical structure factors were computed from previously obtained wave functions using the XFAC option in Crystal14. For each computation, a set of unique Miller indices was firstly generated using the segment description approach (Le Page & Gabe, 1979; Flack, 1984) up to the $sin\theta/\lambda = 1.30\ \text{Å}^{-1}$ reciprocal resolution.

Multipole refinements against theoretical structure factors were performed using the MoPro Suite software package with the analogous strategy as in the case of experimental structure factors. The scale factor and the x, y, z parameters were not refined. Basic statistical descriptors of the refinement are given in the Table 2 and more information can be found in SI, Figures S5 – S12.

2.4. Hirshfeld Surface and QTAIM analyses

Hirshfeld surface analyses (Spackman & McKinnona, 2002; McKinnon *et al.*, 2007) were performed using Crystal Explorer ver. 3.3 (Wolff *et al.*, 2012). The Hirshfeld surfaces and 2D fingerprint plots were generated for each molecule separately, applying their formal charge at level of theory HF/6-31G** (Hehre *et al.*, 1972) using Gaussian09 (Frisch *et al.*, 2016) for experimental geometries (hydrogen atoms positions were not standardized).

A QTAIM analysis was carried out on all experimental, UBDB and theoretical from periodic quantum mechanics electron density models of CC, ACH and GDC crystals. For models in the multipole representation (experimental, UBDB and the one fitted to theoretical structure factors) integrated charges were computed using the WinXPRO program (Stash & Tsirelson, 2002; Stash & Tsirelson, 2007). For exact (not approximated by the multipolar model) theoretical crystal electron densities, integrated charges were calculated for experimental and optimized geometry using TOPOND14 (Gatti *et al.*, 1994; Tsirelson, 2002).

2.5. Electrostatic potentials

Electrostatic potentials for single molecules, selected dimers and entire crystals were computed from charge density models in multipole representation (experimental, UBDB and the one fitted to theoretical structure factors) with the use of the XDPROP module of the XD2016 package (Volkov *et al.*, 2016). For calculations of electrostatic potentials of entire crystals contributions from atoms from at least 3x3x3 unit cells were taken into account. To plot molecular electrostatic potentials on molecular electron isodensities the MoleCoolQt Revision 576 program (Hubschle & Dittrich, 2011) was used.

2.6. Intermolecular interaction energies and electrostatic contributions to them

To compute intermolecular interaction energies for isolated dimers with geometry as found in studied crystals the DFT-based symmetry-adapted perturbation theory (DFT-SAPT) (Jansen & Hesselmann, 2001; Williams & Chabalowski, 2001; Jeziorski *et al.*, 1994) method was applied. Formal charges were assigned to particular molecules (+1 e for cytosinium and adeninium, +2 e for guaninium, 0 e for water molecules and -1 e for chloride anions). Total intermolecular interaction energy in the DFT-SAPT is given as the sum of the first (E^1) and second-order (E^2) perturbation energy terms and the δE^2_{HF} energy term, specifically electrostatic (E^1_{Pol}), induction (E^2_{Ind}) and dispersion (E^2_{Disp}) energy terms together with exchange-repulsion ($E^1_{Exch}, E^2_{Exch-Ind}$ and $E^2_{Exch-Disp}$) terms:

$$E_{tot} = E^1_{Pol} + E^1_{Exch} + E^2_{Ind} + E^2_{Exch-Ind} + E^2_{Disp} + E^2_{Exch-Disp} + \delta E^2_{HF} \quad (3)$$

The corrections δE^2_{HF} were applied in all cases to estimate the polarization effect beyond the second order. DFT-SAPT calculations applied Kohn-Sham (KS) orbitals which were determined using the PBE0AC exchange-correlation (XC) potential (Heßelmann & Jansen, 2002) which consists of using the PBE0 functional with a hybrid kernel consisting of 75% adiabatic local density approximation and 25% coupled Hartree-Fock to solve coupled-perturbed static and frequency-dependent KS equations for the second-order contributions (Gross *et al.*, 1996). For neutral and cationic molecules, the shift parameter (Δ_{xc}) to correct the asymptotic behaviour of the functional was calculated as the difference between the HOMO energy of each monomer (ϵHOMO) and the true ionization potential obtained from the calculation of its neutral/cationic and ionized forms (E_{ion}):

$$\Delta_{xc} = \epsilon HOMO - (-E_{ion}). \quad (4)$$

The values of HOMO and ionization energies were calculated using the PBE0 functional. The anionic systems (chloride anions) were left without asymptotic correction since the XC potentials in these cases are short-ranged and decay with higher powers of 1/r (Lee & Burke, 2010). All DFT-SAPT and HOMO energy calculations were done in the MOLPRO2012.1 program (Werner *et al.*, 2012) with the aug-cc-pVTZ Dunning basis set (Kendall *et al.*, 1992; Dunning, 1989).

For the calculation of electrostatic, polarization, dispersion and repulsion contributions to lattice energy, the semi-classical density sum (the PIXEL method) (Gavezzotti, 2011) was used, which also relays on dimers approximation. Molecular electron densities of molecules bearing their formal charges were obtained using the GAUSSIAN09 revision B.01 program (Frisch *et al.*, 2016) using the 6-31** basis set (Hariharan & Pople, 1973) at the MP2 level of theory. The electron densities were then analysed using the PIXELc module (Gavezzotti, 2003c; Gavezzotti, 2003d; Gavezzotti, 2003a; Gavezzotti, 2003b) of the Coulomb-London-Pauli (CLP) program (Gavezzotti, 2011) which allows the calculation of lattice energies. The total intermolecular interaction energy

was defined in PIXEL as following:

$$E_{tot} = E_{es} + E_{pol} + E_{disp} + E_{rep} \quad (5)$$

where, E_{es} is the electrostatic interaction energy, E_{pol} is the polarization energy, $E_{disp}$is the dispersion energy and E_{rep} repulsion energy between interacting molecules. Due to limitation of the program, *i.e.*it was possible to perform calculation of crystal cohesive energy only for systems which have maximum two molecules in the asymmetric unit, crystal cohesive energy was computed only for the CC structure.

Electrostatic contributions to intermolecular interaction energies and to crystal cohesive energies were also computed from experimental, UBDB and theoretical multipolar models of charge densities with the XDPROP module of the XD2016 package (Volkov *et al.*, 2016). The exact electrostatic energy (E_{es}) was computed with the use of the EPMM method with the distance at which EP is switched to aMM (*rCrit1*) set to 5.0 Å, and the distance at which aMM is switched to mMM (*rCrit2*) set to 100 Å. Default values of the grid size for numerical Coulomb integration were used. The electrostatic energies from molecular monopole moments (charges, E^{q}_{mtp}) were obtained by extracting monopole-monopole contribution from calculations with the mMM option or, alternatively, computed directly from the Coulomb's law: $E_{Coul} = q_1 q_2 / 4\pi\varepsilon_0 r$. For dimer electrostatic energies the INTEREN option was used, whereas for electrostatic contributions to crystal cohesive energies the LATEN option was used with *radii* set to 0 and 100. To differentiate between results for dimers and results for the whole crystal, energies for the latter will be abbreviated as: $E_{coh,es}$ and $E^{q}_{coh,mtp}$.

It is to be noted that E^{1}_{Pol} from the Eq. 3 refers to the exact electrostatic interaction energy and from now onward will be abbreviated as E_{es}.

In the case of refinement against static theoretical structure factors, high negative peaks at the vicinities of nuclei positions are observed on residual maps. This is very well know phenomenon and is usually attributed to limitations of multipole model, which is not flexible enough to accommodate all details of electron density changes while going from spherical isolated atoms/ions into molecules and crystals. To resolve the problem, extended Hansen-Coppens model most often is used in which core electron density term is refined in addition to valence spherical and deformation electron density. However there is another possibility, in our opinion. We have considerable experience with refinement of standard Hansen-Coppens model against valence-only theoretical structure factors; the refinement is a part of procedure for adding a new atom type to the UBDB. Despite the fact that these structure factors do not include information about core electron densities, high-residual peaks close to nuclei positions are still present. Thus, in our opinion, in organic crystals, more elaborated model of spherical part of valence density is necessary. For example, to account for s and p hybridization one can split spherical valence density into two terms, one build form s-orbitals and another one from p–orbitals, and refine populations and expansion-contraction parameters for each term separately. In addition, high residual peaks in the vicinity of chloride nuclei could come from the fact that multipolar models in this study were built on the basis of the SCM electron densities which take into account relativistic effects, whereas during periodic calculations relativistic effects were not included. There is one more possible reason for the presence of residual peaks close to nuclei, it is the inherit difference between Slater and Gaussian functions, the former used in multipole model, the later in Crystal14 calculations.

To be sure that molecular charges (both, these computed from Pval, and these from topological analysis)

obtained from the fit of standard multipole model to theoretical structure factors, we did various extended multipole model modelling for cytosinium chloride, among others: (a) core refinement: in addition to valence density parameters (second and third term of standard Hansen-Coppens model), we refined also expansion-contraction parameters and electron population for K-shell (for Cl-, O, N and C) and L-shell (for Cl-) electron densities; (b) split-valence refinement: in which we modified only the second term of the model, i.e. spherical valence electron density, by splitting it into two spherical terms, one build from s-orbitals and the second one from p-orbitals; (c) second-monopole refinement: in which in addition to standard parameters, we refined populations of monopole functions from valence deformation part of the model together with their individual expansion-contraction parameters for Cl-, O, N and C atoms; (d) split-valence refinement with addition of second monopole for Cl- atom; (e) split-valence refinement with the use of Clementi-Roetti (CR) databank instead of the SCM; (f) refinements against structure factors computed to higher than 1.3 $Å^{-1}$ resolution. All the refinements lead to significant reduction of residual minima and it was very hard to decide which model fits to theoretical structure factors better on the basis of fit statistics alone. Apparently for this case, refinement procedure alone, without any external physical constrains build in the model or in refinement procedure directly, does not allow to find unique description of electron density so close to the nuclei positions. The only way to judge which model reconstructs original electron density (as computed directly from periodic wave function) the best, is comparison of properties. Here we focused on topological charge. The molecular charges computed from refined electron populations from the above refinements were oscillating either around +/-0.80 e or +/- 1.05 e. Similar was for topological charges. Since topological charges computed from direct analysis of theoretical wave function of cytosinium chloride were equal to +/- 0.80 e, we concluded that (a) all refined models are methodically justified and fit equally good theoretical structure factors thus it is impossible to distinguish which model is the best solely on the basis of the fit statistic; (b) it is possible to extend multipole model in such a way as it accounts for residual densities at nuclei vicinity and at the same time reconstructs original electron density in such a way that topological charges are still properly reconstructed, (c) refinement of standard Hansen-Coppens model leads to molecular charges similar to the ones from refinement of more flexible multipole model which reconstruct topological charges of original electron density the best, (+/-0.86 e vs. +/-0.80 e), (d) standard Hansen-Coppens model is good enough (not worse than extended Hansen-Coppens model) to draw the conclusions presented in the paper.

Two examples of refinements which lead to molecular charges around +/-0.80 and one example of refinement which gives charge around +/- 1.05 e are given in Figure S14.

2.7. Transition state search

For select two dimers of protonated nucleobases transition state search was performed in Gaussian16 (Frisch *et al*., 2016). All the geometry optimizations, transition state searches and vibrational frequency analysis were performed with the hybrid B3LYP functional using 6-311G(d,p) basis set. The B3LYP method has repeatedly been shown to yield results that are at least equal to MP2 calculations (Singleton et al., 1997). To properly describe dispersion interactions, Becke–Johnson damping function approach DFT-D3(BJ) was used (Grimme et al., 2011).

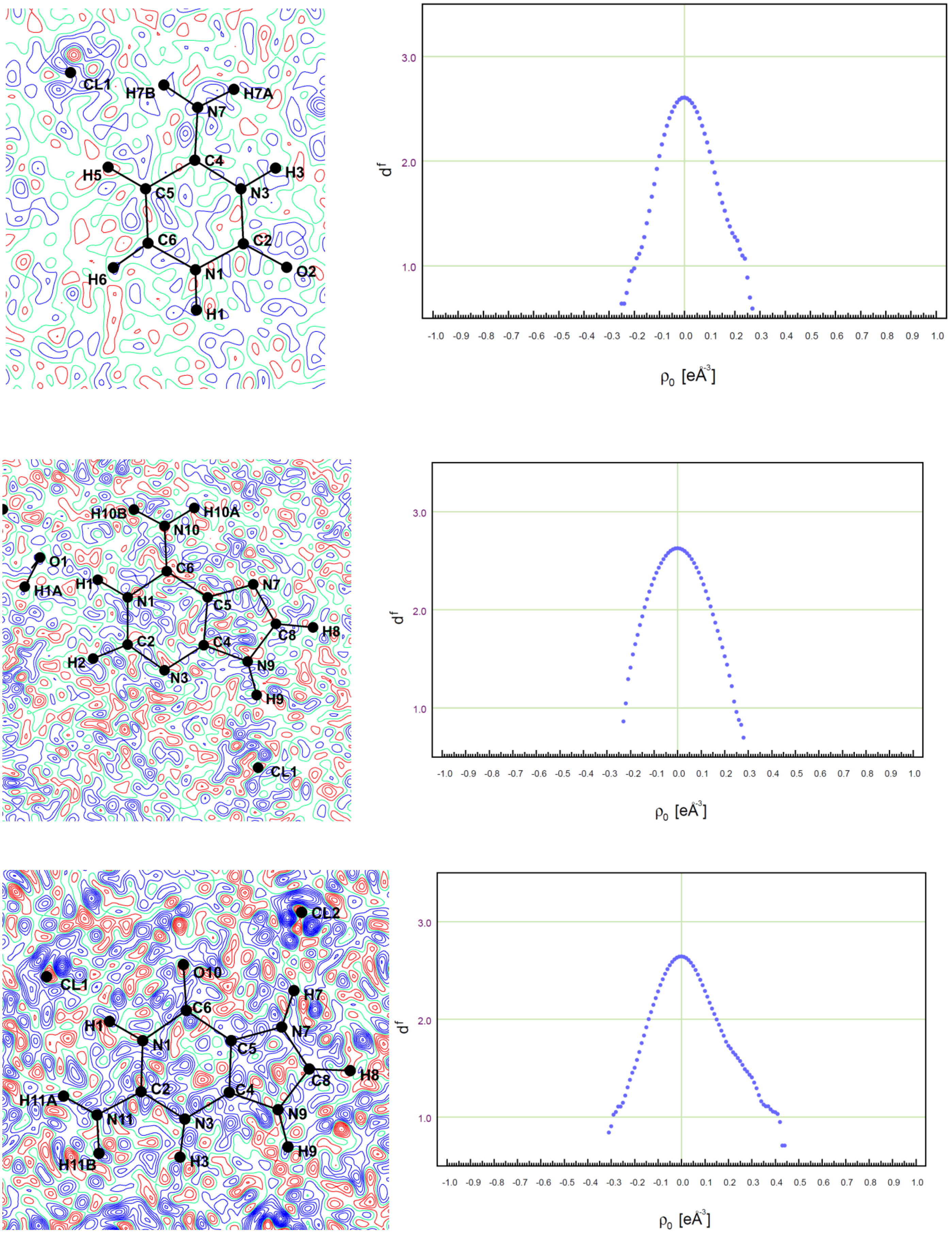

Figure S1

Fourier residual density maps in the plane of nucleobase (right column, contour level: ± 0.05 eÅ^{-3}) and fractal dimension plots of residual density for CC (up), ACH (middle) and GDC (bottom) multipolar refinements against **experimental** structure factors with the use of Cl^{-1} **ionic** scattering radial functions.

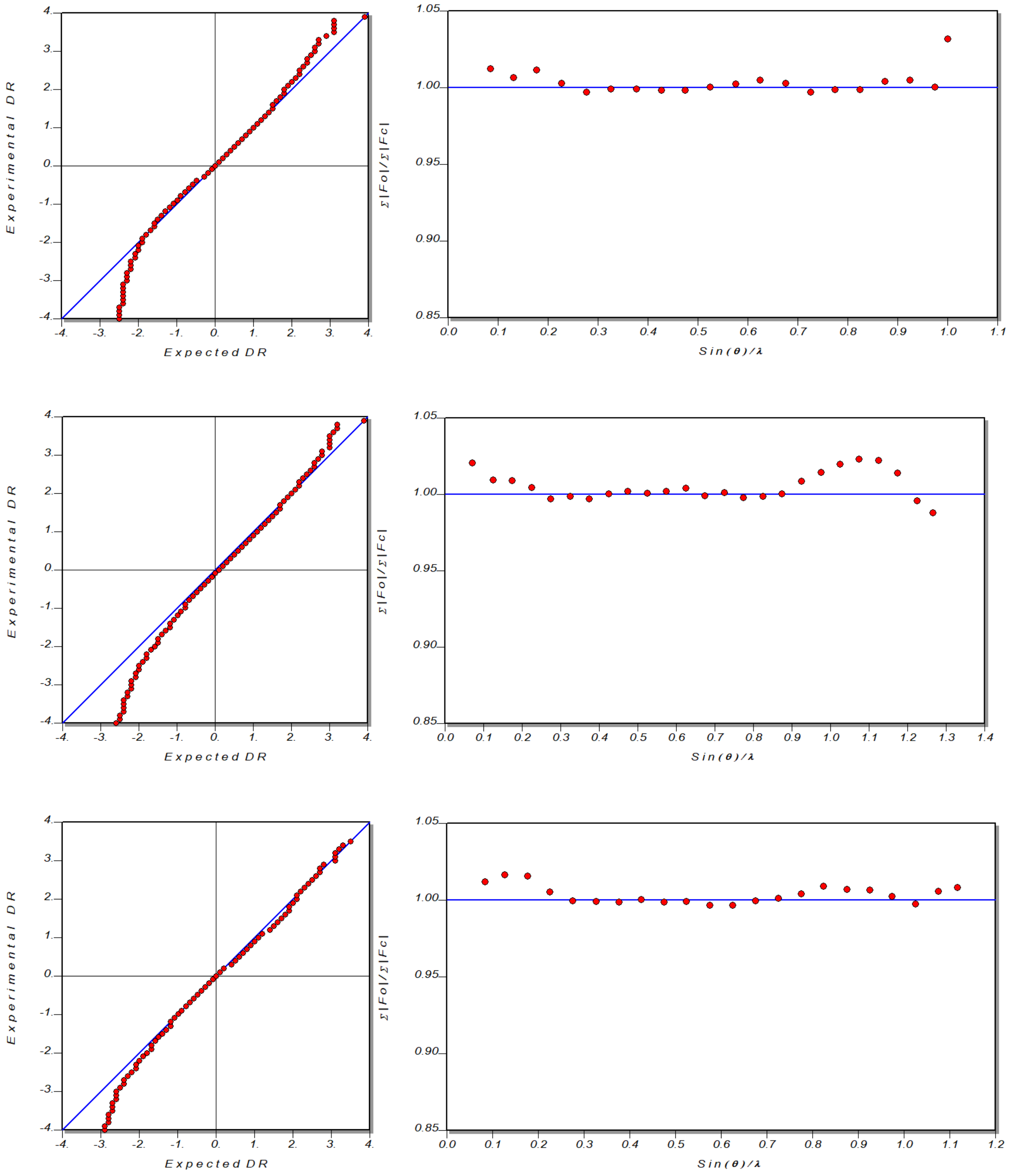

Figure S2

Normal probability plots on F^2 (left) and scale factors, $\sum|F_o|/\sum|F_c|$, with respect to resolution (Å^{-1}) plots (right) for CC (up), ACH (middle) and GDC (bottom) multipolar refinements against **experimental** structure factors with the use of Cl^{-1} **ionic** scattering radial functions. Prepared with the program *XDRKplot* from the *WinGX* package.

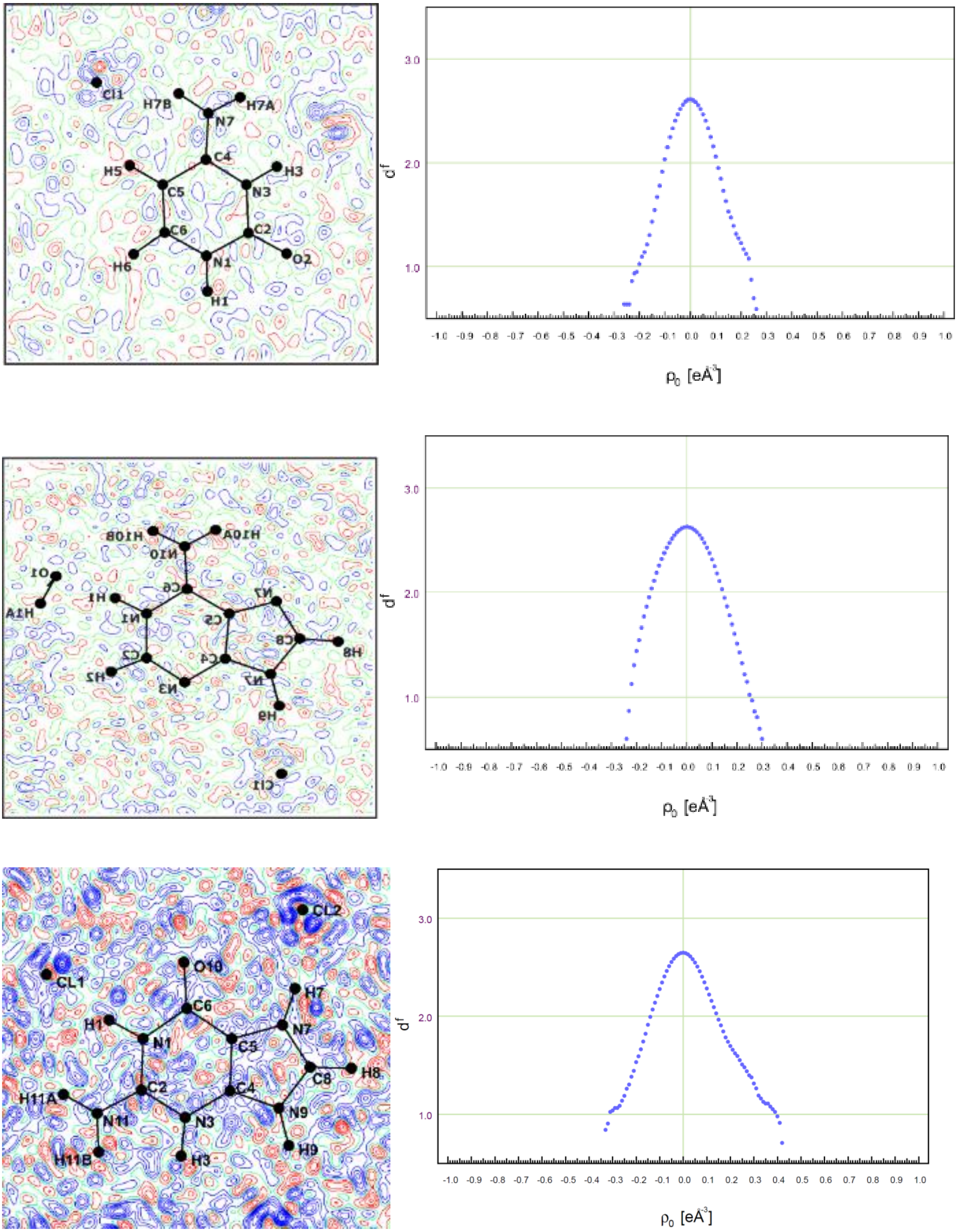

Figure S3

Fourier residual density maps in the plane of nucleobase (right column, contour level: ± 0.05 eÅ^{-3}) and fractal dimension plots of residual density for CC (up), ACH (middle) and GDC (bottom) multipolar refinements against **experimental** structure factors with the use of Cl^0 **neutral** scattering radial functions.

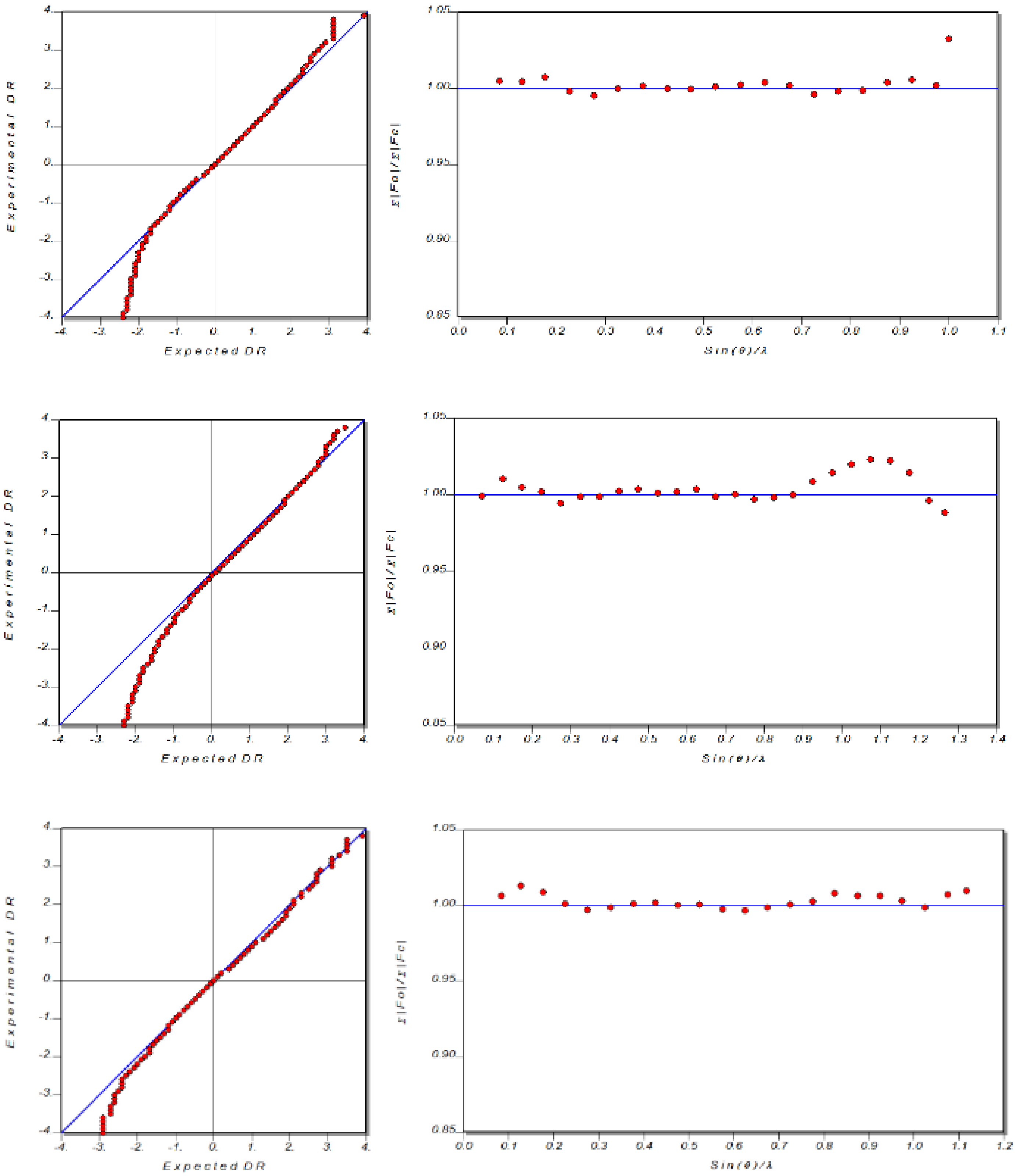

Figure S4
Normal probability plots on F^2 (left) and scale factors, $\sum|F_o|/\sum|F_c|$, with respect to resolution (Å^{-1}) plots (right) for CC (up), ACH (middle) and GDC (bottom) multipolar refinements against **experimental** structure factors with the use of Cl^0 **neutral** scattering radial functions. Prepared with the program *XDRKplot* from the *WinGX* package.

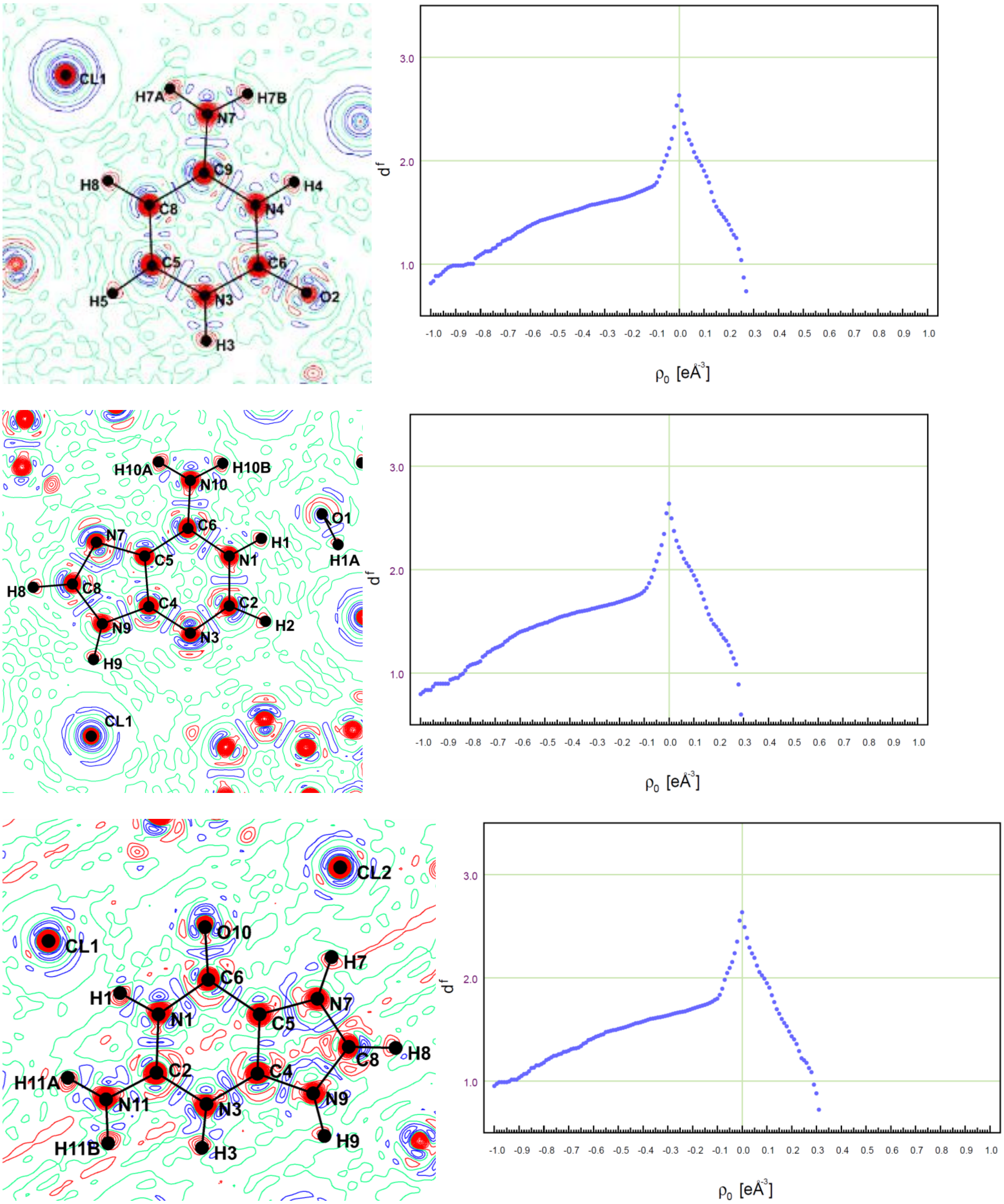

Figure S5

Fourier residual density maps in the plane of nucleobase (right column, contour level: ± 0.05 eÅ^{-3}) and fractal dimension plots of residual density for CC (up), ACH (middle) and GDC (bottom) multipolar refinements against **theoretical structure factors** computed for **experimental geometry** with the use of Cl^{-1} **ionic** scattering radial functions.

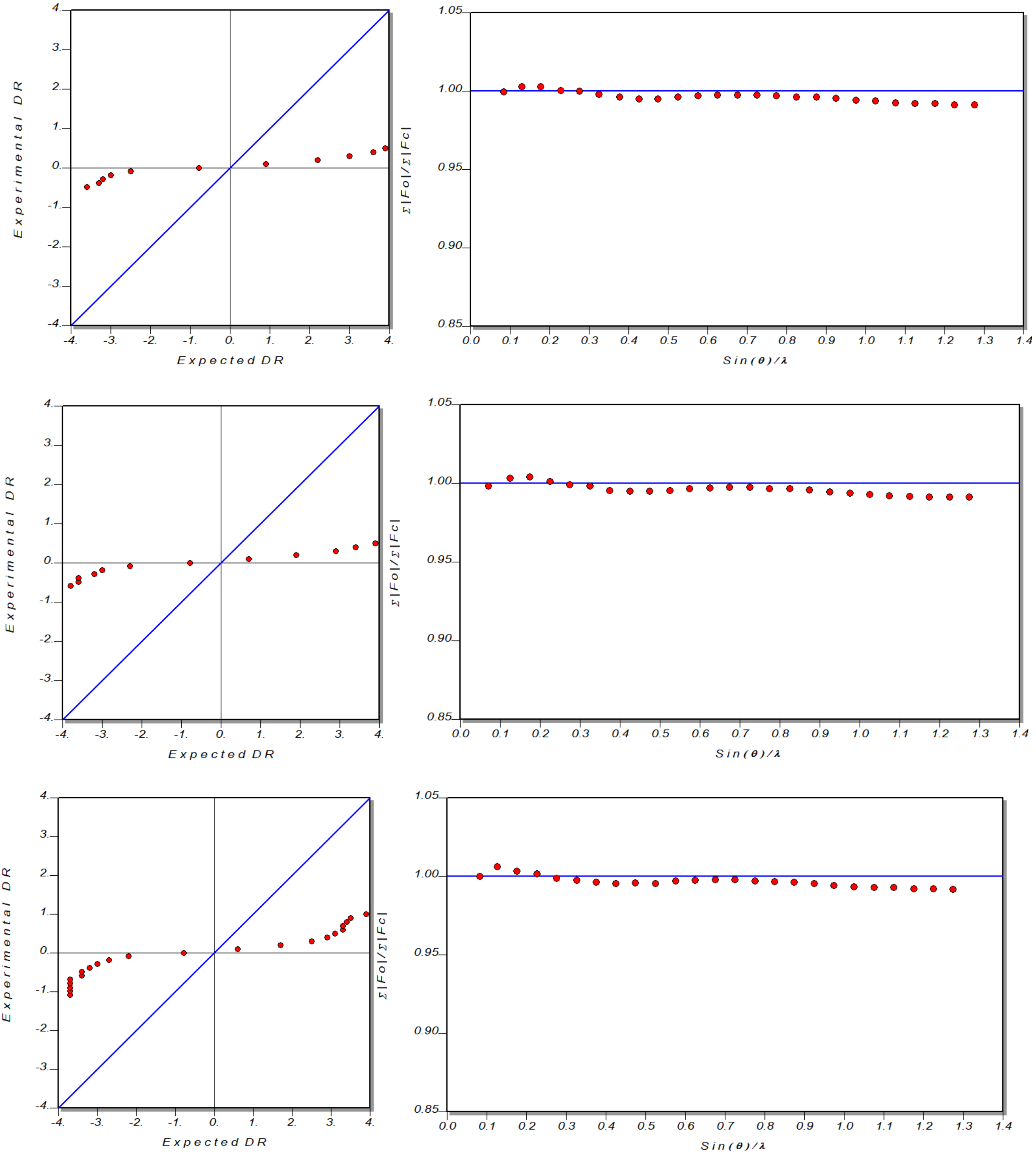

Figure S6

Normal probability plots on F^2 (left) and scale factors, $\sum|F_o|/\sum|F_c|$, with respect to resolution (Å^{-1}) plots (right) for CC (up), ACH (middle) and GDC (bottom) multipolar refinements against **theoretical structure factors** computed for **experimental geometry** with the use of Cl^{-1} **ionic** scattering radial functions. Prepared with the program *XDRKplot* from the *WinGX* package.

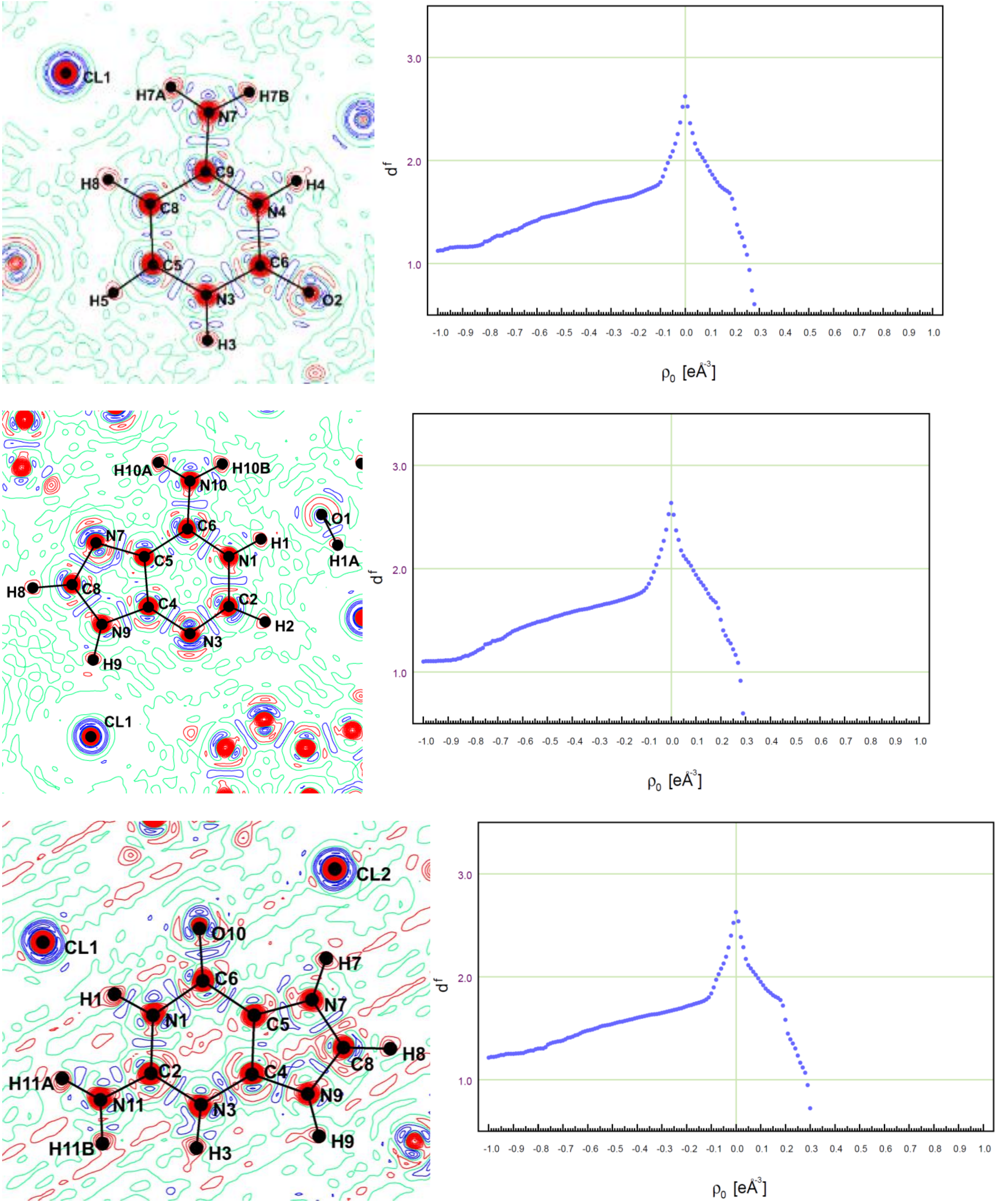

Figure S7

Fourier residual density maps in the plane of nucleobase (right column, contour level: ± 0.05 eÅ^{-3}) and fractal dimension plots of residual density for CC (up), ACH (middle) and GDC (bottom) multipolar refinements against **theoretical structure factors** computed for **experimental geometry** with the use of Cl^0 **neutral** scattering radial functions.

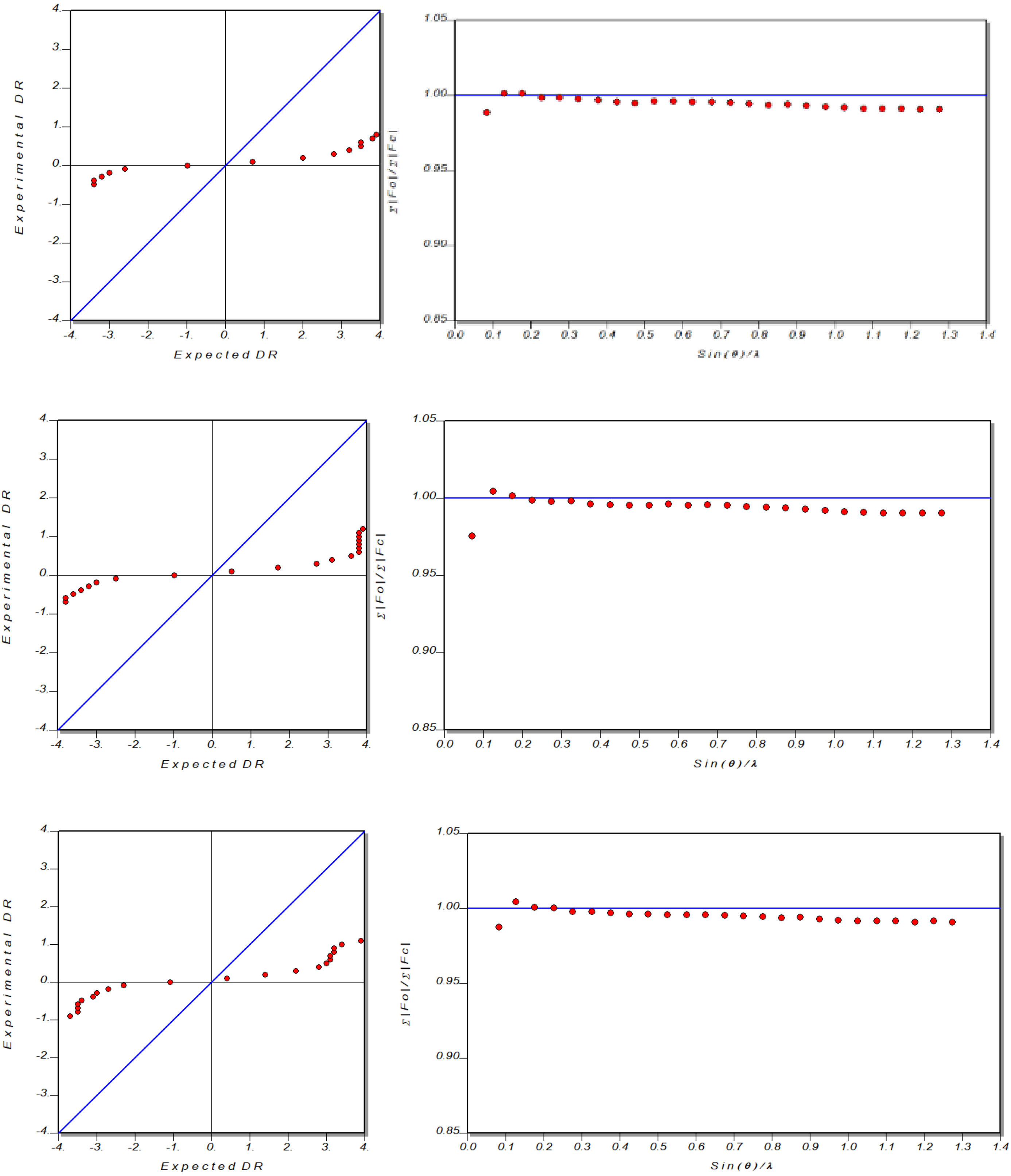

Figure S8

Normal probability plots on F^2 (left) and scale factors, $\sum|F_o|/\sum|F_c|$, with respect to resolution ($Å^{-1}$) plots (right) for CC (up), ACH (middle) and GDC (bottom) multipolar refinements against **theoretical structure factors** computed for **experimental geometry** with the use of Cl^0 **neutral** scattering radial functions. Prepared with the program *XDRKplot* from the *WinGX* package.

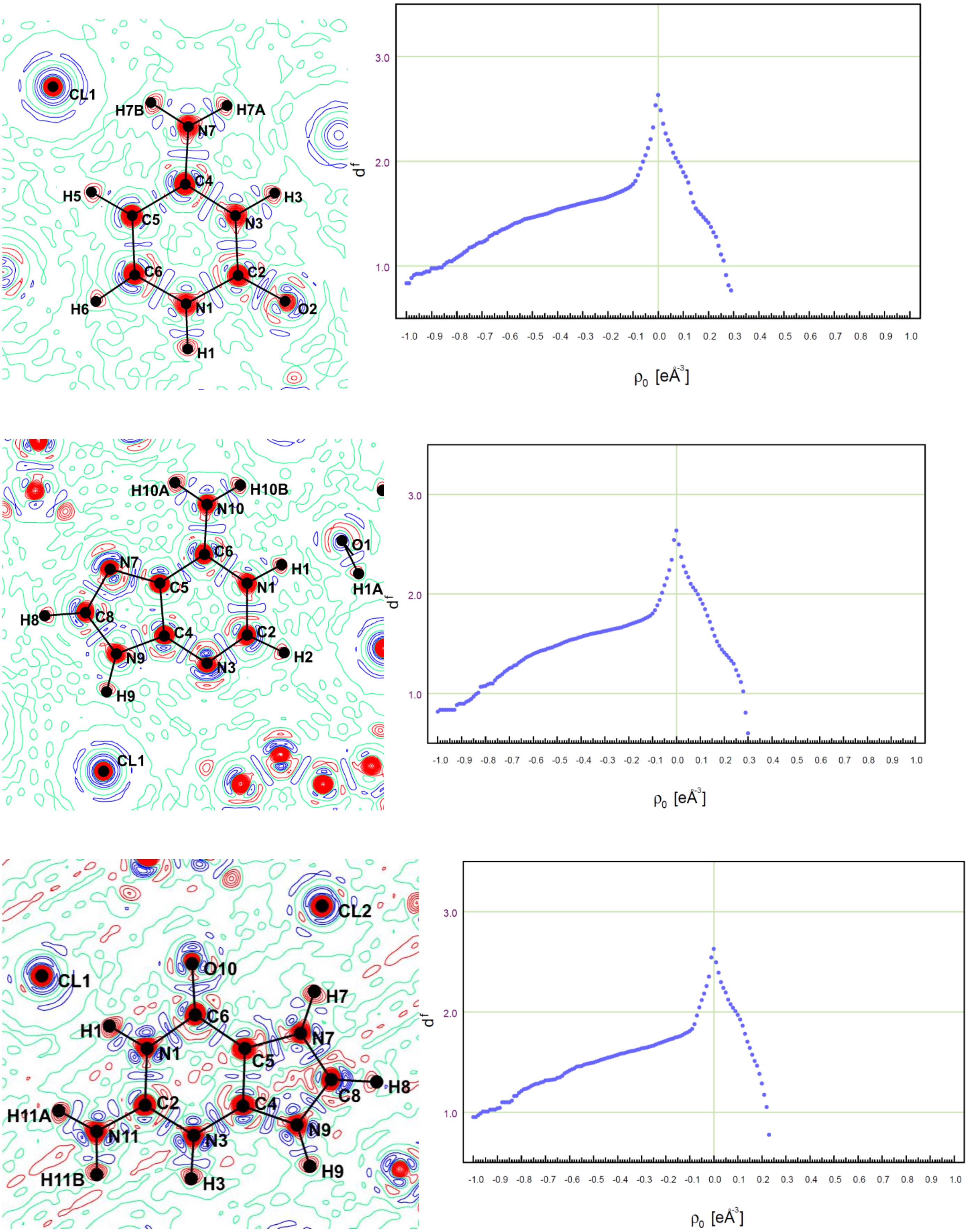

Figure S9

Fourier residual density maps in the plane of nucleobase (right column, contour level: ± 0.05 eÅ^{-3}) and fractal dimension plots of residual density for CC (up), ACH (middle) and GDC (bottom) multipolar refinements against **theoretical structure factors** computed for **optimized geometry** with the use of Cl^{-1} **ionic** scattering radial functions.

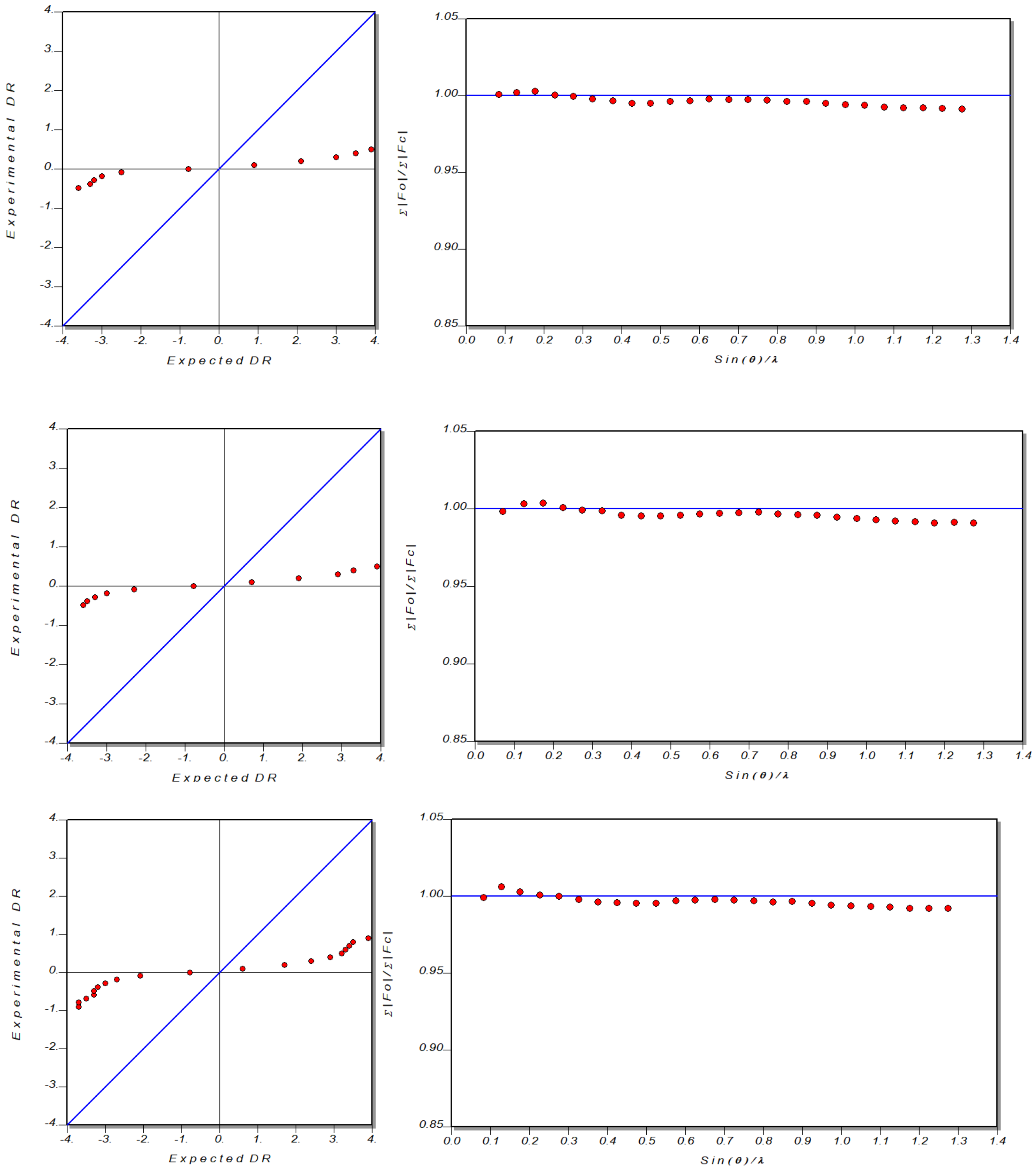

Figure S10

Normal probability plots on F^2 (left) and scale factors, $\sum|F_o|/\sum|F_c|$, with respect to resolution (Å^{-1}) plots (right) for CC (up), ACH (middle) and GDC (bottom) multipolar refinements against **theoretical structure factors** computed for **optimized geometry** with the use of Cl^{-1} **ionic** scattering radial functions. Prepared with the program *XDRKplot* from the *WinGX* package.

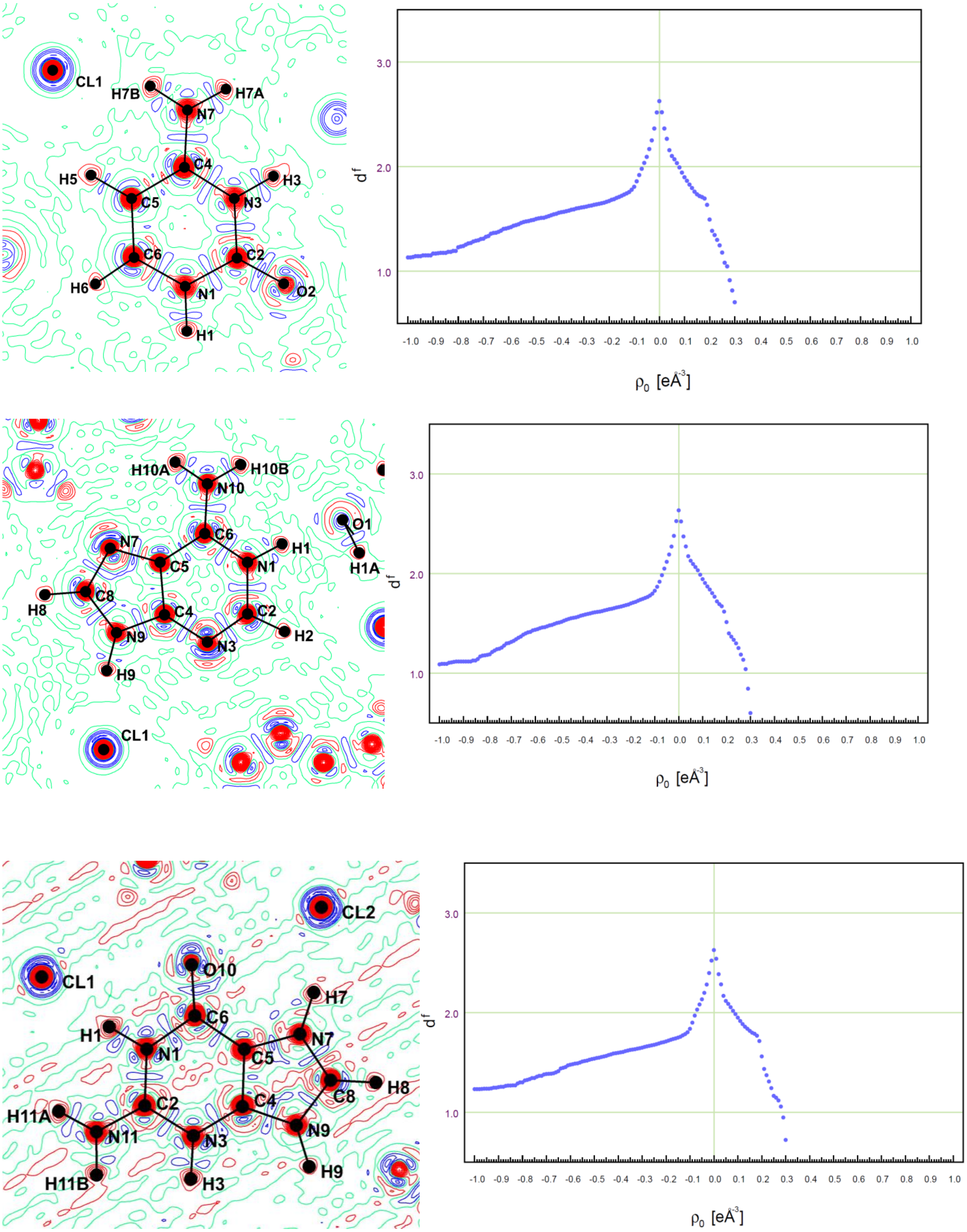

Figure S11

Fourier residual density maps in the plane of nucleobase (right column, contour level: ± 0.05 $eÅ^{-3}$) and fractal dimension plots of residual density for CC (up), ACH (middle) and GDC (bottom) multipolar refinements against **theoretical structure factors** computed for **optimized geometry** with the use of Cl^0 **neutral** scattering radial functions.

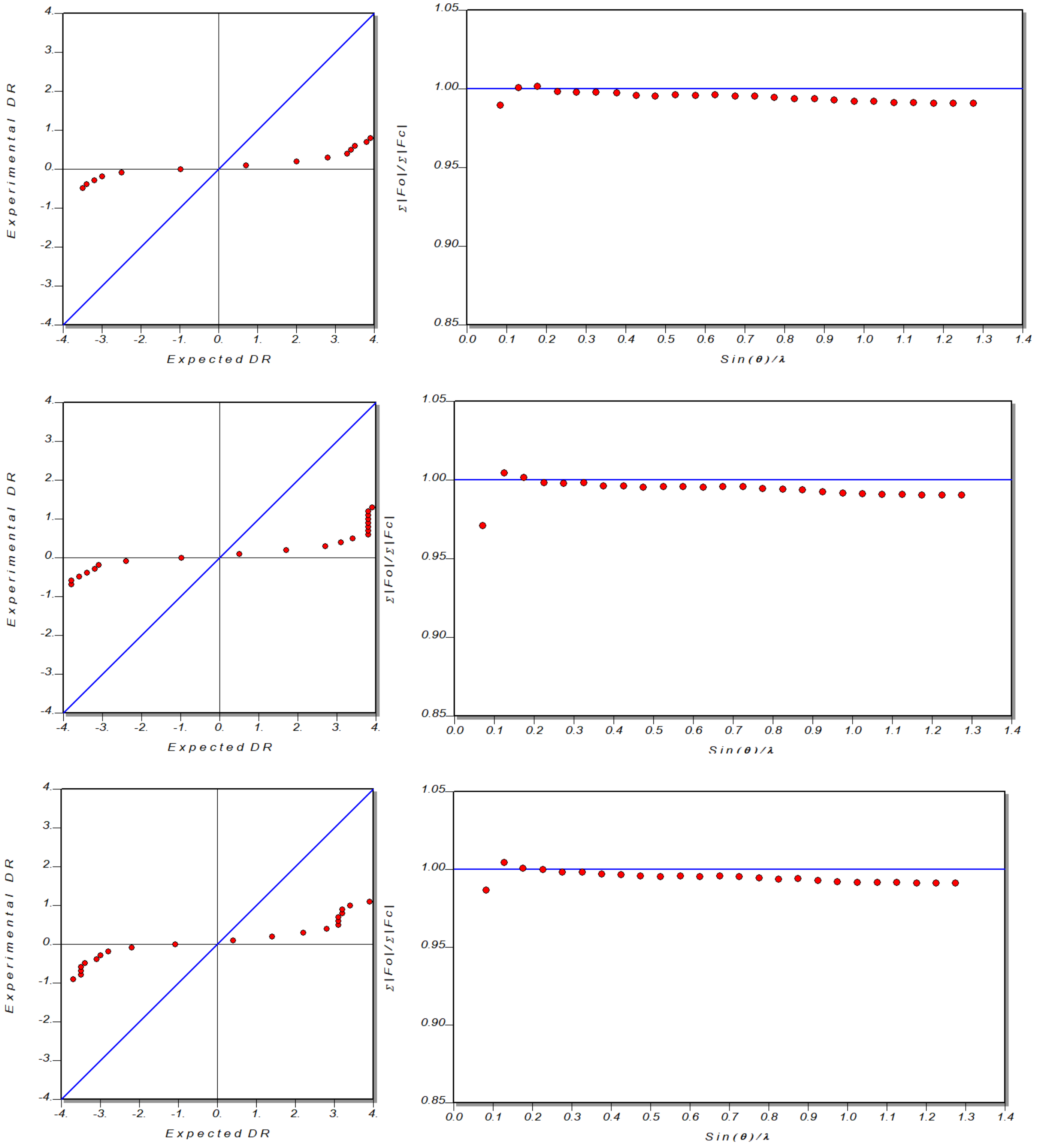

Figure S12

Normal probability plots on F^2 (left) and scale factors, $\sum|F_o|/\sum|F_c|$, with respect to resolution ($Å^{-1}$) plots (right) for CC (up), ACH (middle) and GDC (bottom) multipolar refinements against **theoretical structure factors** computed for **optimized geometry** with the use of Cl^0 **neutral** scattering radial functions. Prepared with the program *XDRKplot* from the *WinGX* package.

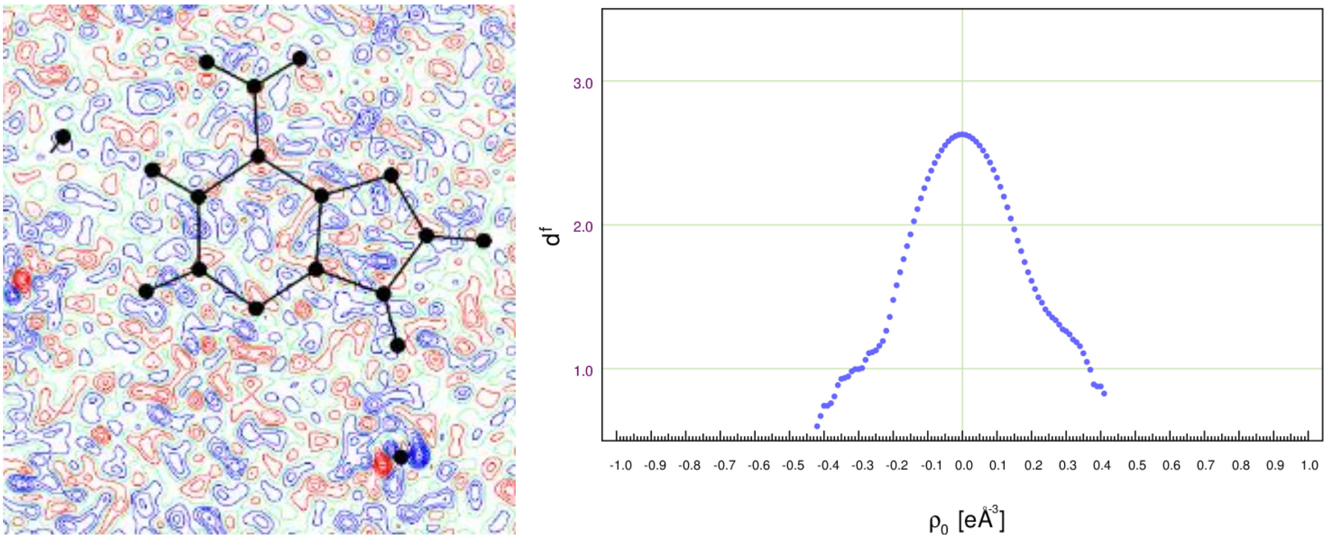

Figure S13

Fourier residual density map in the plane of adenine (right column, contour level: ± 0.05 eÅ^{-3}) and fractal dimension plot of residual density for ACH multipolar refinements against **experimental** structure factors with the use of Cl^{-1} **ionic** scattering radial functions and with **harmonic** approximation of thermal motion for the Cl atom.

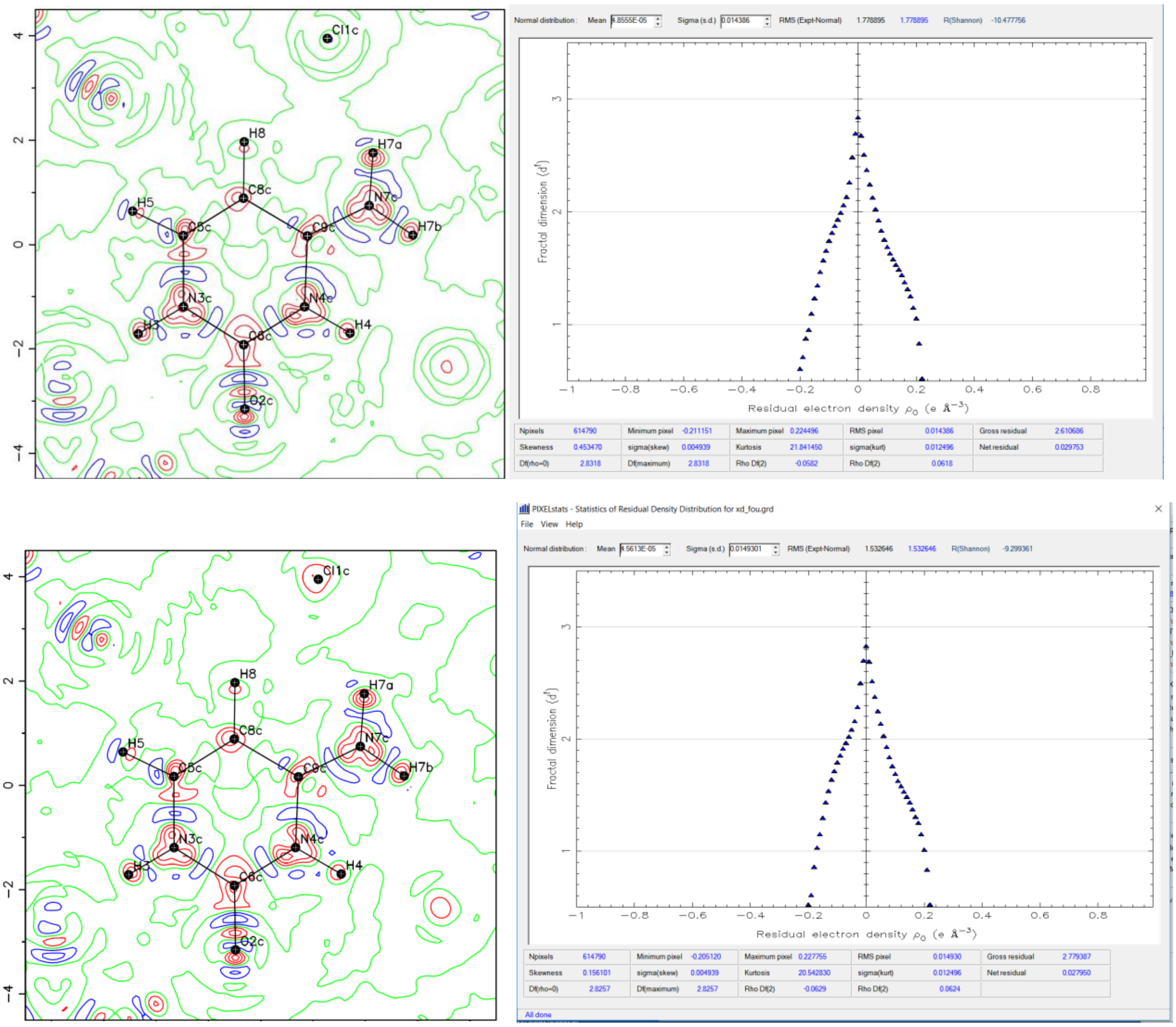

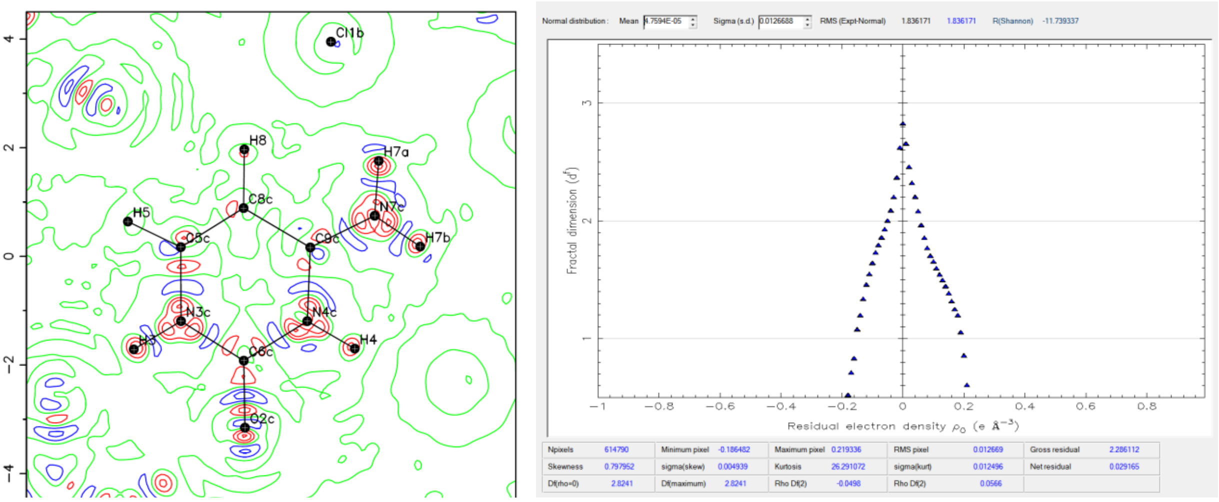

Models:	
Split-valence refinement with M00 for Cl- (top)	for Cl-, O, N and C atoms $P_{val}\kappa^3\rho_{val}(\kappa r)$ term was split to $P_{s-val}\kappa_{s-val}{}^3\rho_{s-val}(\kappa_{s-val}r)$ and $P_{p-val}\kappa_{p-val}{}^3\rho_{p-val}(\kappa_{p-val}r)$; $P_{s-val}, \kappa_{L-cores-val}, P_{p-val}, \kappa_{p-val}$ parameters as well as P_{00} with its own κ_0 for Cl- atom were refined in addition to standard parameters; $\kappa_{s-val}, \kappa_{p-val}, \kappa_0$ for Cl- atom were constrained to equal each other; the SCM databank was used.
Split-valence refinement (middle)	for Cl-, O, N and C atoms $P_{val}\kappa^3\rho_{val}(\kappa r)$ term was split to $P_{s-val}\kappa_{s-val}{}^3\rho_{s-val}(\kappa_{s-val}r)$ and $P_{p-val}\kappa_{p-val}{}^3\rho_{p-val}(\kappa_{p-val}r)$; $P_{s-val}, \kappa_{L-cores-val}, P_{p-val}, \kappa_{p-val}$ parameters were refined in addition to standard parameters; the CR databank was used.
Core refinement (bottom)	for Cl- atom $\rho_{core}(r)$ term was split to $\rho_{K-core}(r)$ and $P_{L-core}\kappa_{L-core}{}^3\rho_{L-core}(\kappa_{L-core}r)$; for O, N and C atoms $\rho_{core}(r)$ term was replaced by $P_{core}\kappa_{core}{}^3\rho_{core}(\kappa_{core}r)$; $P_{L-core}, \kappa_{L-core}, P_{core}, \kappa_{core}$ parameters were refined in addition to standard parameters; the CR databank was used.

Plots:	top	middle	bottom
Topological molecular charge	Q = +/-0.86 e	Q = +/-0.86 e	Q = +/-1.03 e
N obs/N restr/N par	11286/1/212	11286/1/212	11286/1/212
R(F)(%)	0.31	0.32	0.28
Largest diff. peak/hole eÅ^{-3}	0.22, -0.21	0.23, -0.21	0.22, -0.19

Figure S14

Fourier residual density map in the plane of cytosine (contour level: ± 0.05 eÅ^{-3}), fractal dimension plot of residual density and statistical descriptors for CC **extended** multipolar refinement (see description in the table above) against **theoretical** structure factors computed for **experimental geometry** with the use of Cl^{-1} **ionic** scattering radial functions.

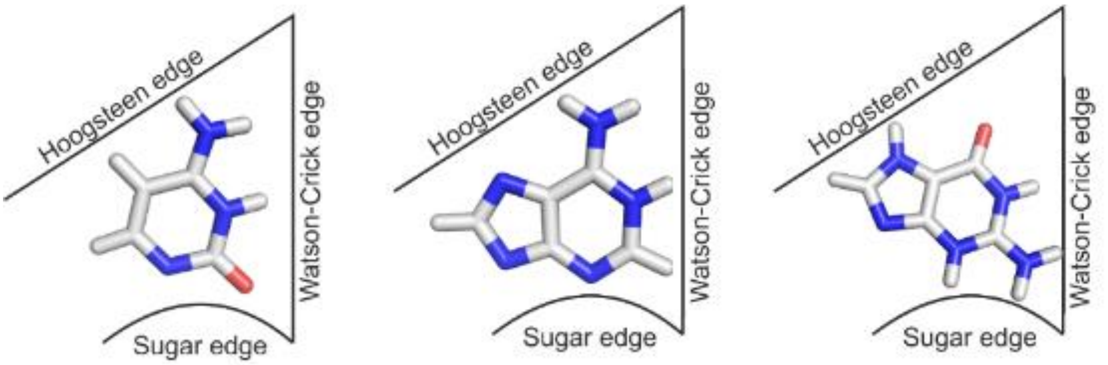

Figure S15

Schematic illustration of the Watson-Crick, Hoogsteen and sugar edges for cytosinium (left), adeninium (middle) and guaninium (right) moieties.

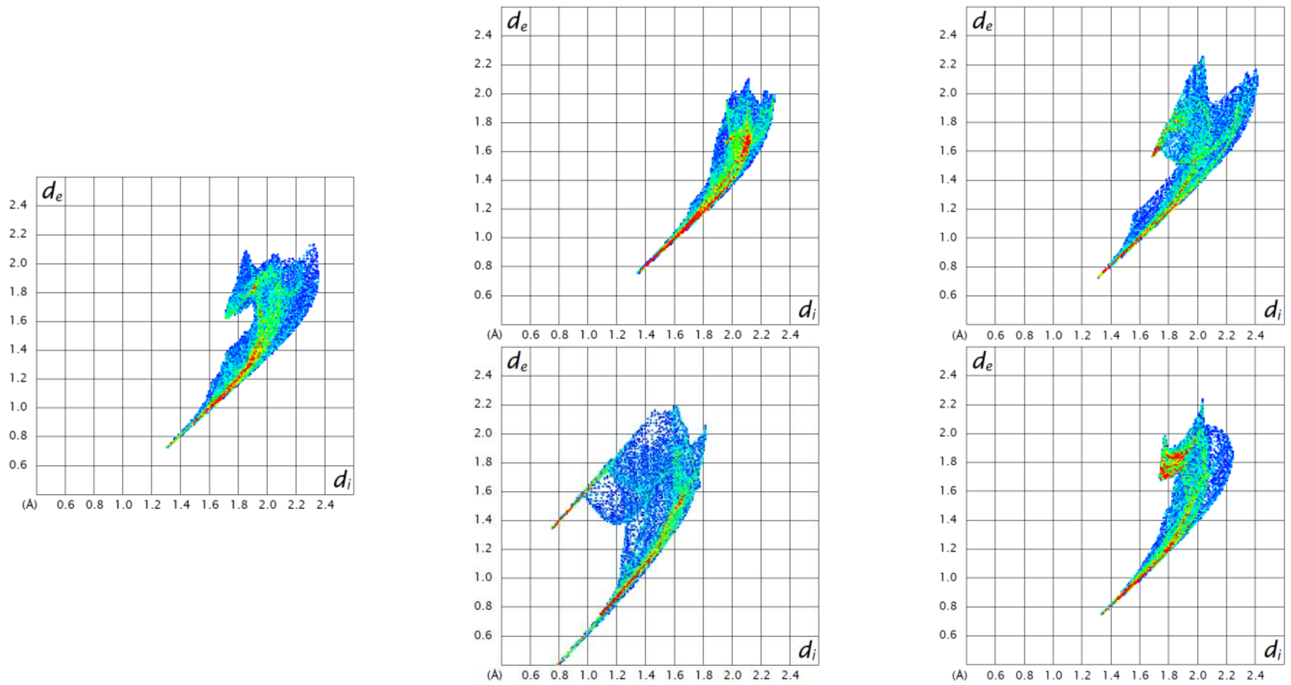

Figure S16

Hirshfeld surface based fingerprints of the chloride anion in the CC (left), the chloride anion (up) and the water molecule (down) in the ACH (middle), and the first (up) and second (down) chloride anions in the GDC (right) crystal structures.

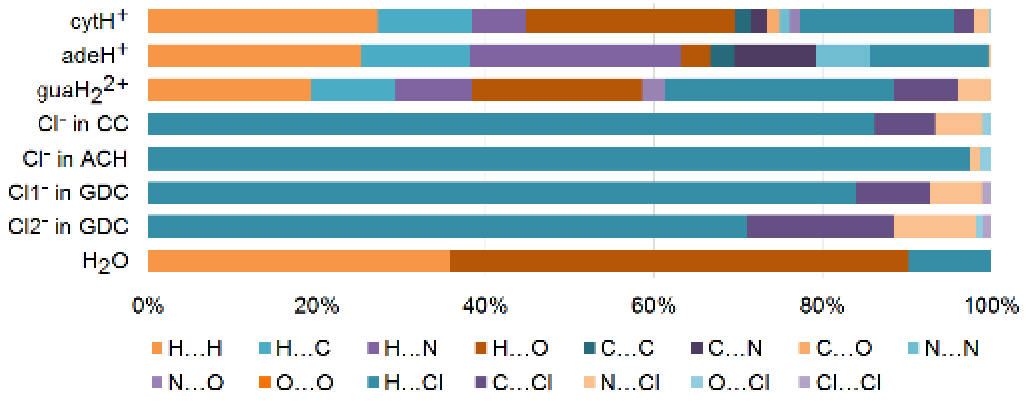

Figure S17

Percentage contributions of different interatomic contact types to the Hirshfeld surfaces of particular molecules present in the CC, ACH and GDC.

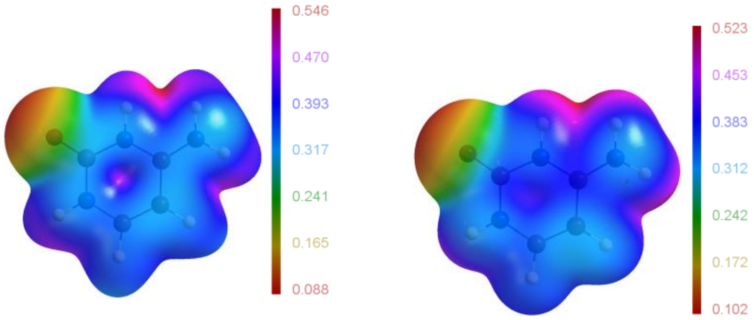

$V^{+}_{av.}$ = 0.342 eÅ^{-1}
π = 0.065 eÅ^{-1}

$V^{+}_{av.}$ = 0.340 eÅ^{-1}
π = 0.066 eÅ^{-1}

Figure S18
Electrostatic potential (eÅ^{-1}) of isolated cytosinium cation bearing formal charge as modeled by UBDB (left) or computed by gas-phase quantum mechanics methods (right, Gaussian16/B3LYP/aug-cc-pVTZ) mapped on respective electron-density isosurfaces at ρ = 0.0135 eÅ^{-3}. $V^{+}_{av.}$ – average of positive surface values, π – average deviation from the average positive surface value (Politzer *et al.*, 2001).

Table S1
Cohesive energy calculations using CRYSTAL14 package for experimental and optimized (in bold) crystal geometry.
The cohesive energy of a molecular crystal is the energy difference between the total energy of the unit cell and the isolated single molecule in the gas phase. It corresponds to the packing energy due to the interaction among the molecules in the crystal. For comparatively rigid molecules (i.e. those having almost similar geometry in gas phase and in crystal) the cohesive energy expression reduces to $E_{Cohesive} = \Delta E_{cond}$ + BSSE with $\Delta E_{cond} = (1/Z)E_{bulk} - E_{mol}$ and $BSSE = E_{mol,bulk} - E_{mol,ghost}$, where E_{bulk} is the total energy of the unit cell and must be referred to the value of Z; $E_{mol,bulk}$ is the energy associated with a single molecule having the same geometry as in the bulk; $E_{mol,ghost}$ is calculated energy of a single molecule by using ghost functions on the surrounding atoms. All calculations were performed at CC/pVDZ level of theory on the experimental and geometry optimized structures. Coordinates (in .cif format) for optimized structures are given below the table.

E_{bulk} (Hartrees)	$E_{mol,bulk}$ (Hartrees)	$E_{mol,ghost}$ (Hartrees)	ΔE_{cond} (kcal mol^{-1})	$BSSE$ (kcal mol^{-1})	$E_{Cohesive}$ (kcal mol^{-1})
Cytosinium chloride					
-3422.416204	-855.604051	-855.882407	-174.67	11.08	-163.59
**-3422.489853**	**-855.622463**	**-855.891594**	**-166.69**	**2.19**	**-164.50**
Adeninium chloride hemihydrate					
-3864.702671	-966.175667	-965.857763	-199.48	25.93	-173.55
**-3864.719060**	**-966.179765**	**-965.864670**	**-197.72**	**24.39**	**-173.33**
Guaninium dichloride					
-5855.666680	-1463.972360	-1463.284228	-431.81	36.18	-395.63
**-5854.408829**	**-1463.602207**	**-1462.850861**	**-471.48**	**36.27**	**-435.21**

```
data_CCopt
_cell_angle_alpha                     90.000000
_cell_angle_beta                      34.385000
_cell_angle_gamma                     90.000000
_cell_length_a                        10.99470000
_cell_length_b                        6.87740000
_cell_length_c                        14.34960000
_cell_volume                          612.779811
_symmetry_space_group_name_H-M        'P 21/C'
_chemical_formula_sum                 'H6 C4 N3 O1 CL1 '

loop_
_atom_site_label
_atom_site_fract_x
_atom_site_fract_y
_atom_site_fract_z
_atom_site_occupancy
CL        0.229422591913      0.123454737097      0.438809887289    1.000000
O         0.365291665971     -0.162090277125      0.012590130639    1.000000
N        -0.286961459101     -0.183726302275     -0.154669250408    1.000000
N        -0.439786382419     -0.433144521696     -0.144909648693    1.000000
C        -0.098816659528     -0.284450076850     -0.268487135685    1.000000
C        -0.462530018640     -0.252031004726     -0.091168057226    1.000000
N        -0.252311525746      0.288412798720     -0.302871307081    1.000000
C        -0.075340599570     -0.461886302580     -0.324181655930    1.000000
```

```
C         -0.255153980015       0.459727324569      -0.259285031276   1.000000
H          0.032407183608      -0.215953806076      -0.313529452742   1.000000
H         -0.114649660205       0.205777651571      -0.387877766812   1.000000
H          0.075141435315       0.457647371066      -0.416468457402   1.000000
H          0.430011307234      -0.484424337023      -0.102413945885   1.000000
H         -0.381748436706       0.239655758792      -0.257930978604   1.000000
H         -0.305179677402      -0.050212007893      -0.110361754287   1.000000

data_ACHopt
_cell_angle_alpha                  90.000000
_cell_angle_beta                   93.526200
_cell_angle_gamma                  90.000000
_cell_length_a                     8.69360000
_cell_length_b                     4.81890000
_cell_length_c                     17.69710000
_cell_volume                       739.991409
_symmetry_space_group_name_H-M    'P 1 2/N 1'
_chemical_formula_sum              'H7 C5 N5 O1 CL1 '

loop_
_atom_site_label
_atom_site_fract_x
_atom_site_fract_y
_atom_site_fract_z
_atom_site_occupancy
CL         0.490904446857      -0.168675735162       0.273549639418   1.000000
O          0.250000000000      -0.250433452527      -0.250000000000   1.000000
H          0.266790750316      -0.370434360492      -0.343186266776   1.000000
N          0.122597019662       0.200349055746       0.432337206637   1.000000
N          0.271936028659      -0.403263070808      -0.401320558744   1.000000
C          0.192600136212       0.374072861931       0.486002483183   1.000000
N          0.381794105874      -0.245198460284       0.487093429249   1.000000
C          0.292561866926      -0.442912074569       0.452293691014   1.000000
C          0.181522978891       0.393205908833      -0.434837006107   1.000000
N          0.282616551724       0.491843966629       0.377166388209   1.000000
N          0.094896187817       0.233718530313      -0.393984998001   1.000000
C          0.366591662371      -0.234543953364      -0.439930471998   1.000000
C          0.180170796801       0.277836241564       0.368070378348   1.000000
H          0.432534753988      -0.083084363265      -0.405328818607   1.000000
H          0.150611542846       0.181338563095       0.313386797315   1.000000
H          0.342286302094      -0.408230854247       0.336116600666   1.000000
H          0.022226731060       0.086563208180      -0.419540899763   1.000000
H          0.161126087896      -0.121938047950      -0.248037717392   1.000000
H          0.084819740446       0.264359540929      -0.337002526874   1.000000

data_GDCopt
_cell_angle_alpha                  90.000000
_cell_angle_beta                   90.000000
_cell_angle_gamma                  90.000000
_cell_length_a                     13.59390000
_cell_length_b                     6.48410000
_cell_length_c                     9.86000000
_cell_volume                       869.101881
_symmetry_space_group_name_H-M    'P N M A'
_chemical_formula_sum              'H7 C5 N5 O1 CL2 '

loop_
_atom_site_label
_atom_site_fract_x
_atom_site_fract_y
_atom_site_fract_z
_atom_site_occupancy
CL        -0.468832257201      -0.250000000000       0.327889684176   1.000000
CL        -0.324885901508      -0.250000000000      -0.000834827311   1.000000
O         -0.353380245938      -0.250000000000      -0.343222692657   1.000000
N         -0.151756644473      -0.250000000000      -0.201420822332   1.000000
H         -0.202655696629      -0.250000000000      -0.121378748952   1.000000
N         -0.185096745492      -0.250000000000       0.252311903560   1.000000
H         -0.249906322668      -0.250000000000       0.198404488598   1.000000
H         -0.119524004352      -0.250000000000       0.199496137715   1.000000
N         -0.092640085280      -0.250000000000       0.450465264591   1.000000
H         -0.029449846159      -0.250000000000       0.392018533043   1.000000
N         -0.016604269194      -0.250000000000      -0.321106681305   1.000000
H          0.057676494444      -0.250000000000      -0.344904166745   1.000000
N         -0.265591588330      -0.250000000000       0.459142227883   1.000000
H         -0.333090693642      -0.250000000000       0.407866709993   1.000000
C         -0.093130323383      -0.250000000000      -0.410859803920   1.000000
C         -0.054452024517      -0.250000000000      -0.194014116859   1.000000
H         -0.010508475081      -0.250000000000      -0.102091132205   1.000000
C         -0.181522298366      -0.250000000000       0.385579624743   1.000000
C         -0.273251218096      -0.250000000000      -0.399322504594   1.000000
C         -0.178457682629      -0.250000000000      -0.336344219379   1.000000
```

Table S2

A list of symmetry operation defining selected dimers in the CC, ACH and GDC structures. To build a given dimer symmetry card assigned to it should be applied to the second molecule in the dimer represented by the second letter in the dimer name (A nucleobase cation, B chloride anion, W water molecule).

Dimer	Symmetry card
Cytosinium chloride (CC)	
AA1	-x, -y+2, -z+1
AA2	-x, -y+1, -z+1
AA3	x+0.5, -y+1.5, z+0.5
AA4	-x+1, -y+1, -z+1
AA5	x, y-1, z
AA6	-x+0.5, y+0.5, -z+1.5
AA7	-x,+1, -y, -z+1
AB1	x, y, z
AB2	-x+1, -y, -z+1
AB3	-x+1, -y+1, -z+1
AB4	x-0.5, -y+0.5, z-0.5
AB5	x, y+1, z
AB6	-x+0.5, +y+0.5, -z+1.5
BB1	-x+1, -y, -z+1
Adeninium chloride hemihydrate (ACH)	
AA1	-x, -y, -z+1
AA2	-x+1, -y+2, -z+1
AA3	-x+1, -y+1, -z+1
AA4	x, y+1, z
AA5	-x, -y+1, -z+1
AA6	-x+0.5, y, -z+1.5
AB1	x-0.5, -y+1, z+0.5
AB2	-x+0.5, y-1, -z+0.5
AB3	x, y, z
AB4	-x+1, -y+2, -z+1
AB5	-x+1, -y+1, -z+1
AB6	x, y-1, z
AB7	-x+0.5, y, -z+0.5
AW1	x, y, z
BW1	x+0.5, -y+2, z-0.5
BW2	-x+1, -y+1, -z+1
BB1	-x+0.5, y, -z+0.5
Guaninium dichloride (GDC)	
AA1	x+0.5, -y+1.5, -z+1.5
AA2	-x+1.5, -y+1, z+0.5
AA3	-x+2, y-0.5, -z+1
AA4	x, y-1, z
AA5	x, y, z-1
AB1	x+0.5, -y+1.5, -z+0.5
AB2	x, y, z
AB3	x+0.5, -y+1.5, -z+1.5
AB4	x+0.5, -y+1.5, -z+1.5
AB5	x, y, z
AB6	x, y, z-1
AB7	-x+1.5, -y+1, z+0.5
AB8	-x+1.5, -y+1, z-0.5
AB9	-x+1, y+0.5, -z+1
BB1	x, y, z-1

Table S3

Components of the *DFT-SAPT* and *PIXEL* interaction energies (kcal mol^{-1}) for the selected dimers extracted from the CC, ASH and GDC crystal structures. For the symmetry operations required to build particular dimers see Table S2. It is to be noted that E^1_{Pol} refers to exact electrostatic energy of interaction between two charge densities of isolated molecules and in the main text of the article is abbreviated as E_{es}.

Dimer	Center of mass distance (Å)	DFT-SAPT								PIXEL				
		E^1_{Pol}	E^1_{Exch}	E^2_{Ind}	$E^2_{Exch-Ind}$	E^2_{Disp}	$E^2_{Exch-Disp}$	δE^2_{HF}	E_{Tot}	E_{es}	E_{pol}	E_{disp}	E_{rep}	E_{Tot}
Cytosinium chloride (CC)														
AA1	6.060	16.6	29.2	-19.0	9.1	-9.3	1.6	-8.2	20.0	19.5	-13.8	-5.6	20.4	20.6
AA2	3.455	61.9	6.2	-6.0	2.0	-8.1	0.7	-1.3	55.4	62.3	-7.3	-6.8	3.9	52.2
AA3	6.739	39.8	4.1	-3.3	1.0	-2.8	0.3	-1.0	38.1	40.3	-2.7	-2.1	2.5	38.0
AA4	5.385	68.5	3.9	-4.9	1.5	-4.3	0.4	-0.8	64.3	69.3	-6.0	-3.8	1.9	61.4
AA5	6.877	50.0	0.4	-2.3	0.1	-1.1	0.0	-1.0	46.2	50.3	-2.8	-1.1	0.1	46.6
AA6	6.740	56.2	0.9	-3.0	0.3	-1.5	0.1	-0.8	52.2	55.9	-3.7	-1.6	0.5	51.0
AA7	9.940	47.2	0.0	-1.2	0.0	-0.1	0.0	-0.7	45.1	47.7	-1.3	-0.2	0.0	46.2
AB1	4.837	-96.8	18.3	-22.5	13.2	-7.3	1.9	-7.8	-101.1	-97.3	-15.8	-4.6	11.6	-106.1
AB2	5.828	-79.7	6.8	-12.3	6.7	-3.1	0.8	-3.7	-84.5	-79.3	-7.2	-2.1	3.3	-85.3
AB3	3.309	-82.8	11.0	-23.0	17.0	-6.7	1.8	-2.6	-85.3	-79.0	-11.4	-3.4	4.9	-88.9
AB4	3.964	-106.2	30.1	-35.8	22.2	-9.9	2.7	-12.0	-108.9	-101.0	-20.5	-5.8	16.9	-110.3
AB5	4.890	-69.7	3.2	-7.9	3.8	-2.3	0.5	-2.5	-74.9	-68.3	-5.5	-1.3	0.9	-74.3
AB6	4.513	-70.2	3.3	-8.6	4.5	-2.6	0.6	-2.3	-75.3	-68.5	-5.8	-1.3	0.8	-74.9
BB1	3.991	80.9	1.7	-7.6	2.8	-1.8	0.6	-1.2	75.4	83.0	-3.8	-0.7	0.8	79.3
Adeninium chloride hemihydrate (ACH)														
AA1	6.076	29.5	24.5	-14.8	7.3	-9.2	1.4	-5.5	33.2	28.4	-14.3	-6.7	19.6	27.0
AA2	6.909	41.4	4.5	-3.2	1.4	-3.7	0.4	-0.7	40.1	41.9	-2.2	-3.0	2.9	39.5
AA3	4.596	55.2	6.3	-5.5	2.5	-7.5	0.7	-1.1	50.7	55.7	-5.0	-6.4	4.3	48.6
AA4	4.819	55.7	5.1	-4.8	2.0	-6.3	0.6	-0.9	51.4	56.5	-4.4	-5.3	3.0	49.8
AA5	4.134	56.3	7.9	-6.7	3.2	-9.4	0.9	-1.3	51.0	57.9	-6.2	-7.9	4.9	48.7
AA6	9.027	53.4	0.0	-2.0	0.0	-0.3	0.0	-1.0	50.0	53.7	-2.4	-0.3	0.0	50.9
AB1	5.666	-86.2	14.4	-18.5	10.2	-5.2	1.4	-7.2	-91.2	-86.4	-12.8	-3.2	9.6	-92.7
AB2	5.927	-61.0	2.7	-7.3	3.4	-2.2	0.4	-1.9	-65.9	-58.6	-6.5	-1.9	3.3	-63.7
AB3	4.917	-81.1	20.0	-24.0	14.4	-6.9	1.9	-9.0	-84.7	-81.2	-17.3	-3.8	14.4	-87.9
AB4	5.749	-72.7	5.5	-9.9	4.4	-3.0	0.7	-3.8	-78.8	-71.9	-7.0	-1.9	2.5	-78.3
AB5	4.787	-76.7	4.5	-11.3	6.1	-3.2	0.7	-2.5	-82.4	-75.4	-7.5	-1.9	1.4	-83.3
AB6	5.561	-57.7	1.8	-5.7	2.3	-1.7	0.3	-1.7	-62.3	-57.0	-4.3	-0.9	0.3	-61.8
AB7	5.328	-59.4	6.5	-9.1	4.4	-3.2	0.7	-3.6	-63.7	-60.0	-5.1	-1.2	0.6	-65.6
AW1	4.752	-14.8	14.5	-8.3	4.6	-4.5	0.9	-3.2	-11.0	-15.9	-7.0	-3.1	10.5	-15.4
BW1	3.031	-18.5	16.1	-13.0	8.8	-5.4	1.6	-3.8	-14.3	-19.0	-9.3	-2.8	10.1	-21.0
BW2	3.659	5.7	1.6	-2.3	1.2	-1.1	0.2	-0.4	4.9	6.6	-1.3	-0.5	0.2	5.0
BB1	4.262	76.6	0.9	-5.4	1.5	-1.2	-1.0	-1.0	70.4	77.8	-2.9	-0.5	0.3	74.8
Guaninium dichloride (GDC)														
AA1	7.637	162.1	7.5	-11.1	2.2	-3.8	0.4	-3.3	154.0	163.7	-10.3	-3.2	6.2	156.4
AA2	6.309	192.5	1.2	-8.9	0.4	-2.5	0.1	-2.1	180.7	191.4	-12.7	-2.5	0.4	176.7
AA3	5.781	239.2	0.6	-10.8	0.2	-2.2	0.0	-3.9	223.2	237.1	-18.4	-2.8	0.2	216.1
AA4	6.484	174.2	0.0	-2.4	0.0	-0.3	0.0	-0.9	170.6	173.6	-5.6	-0.3	0.0	167.7
AA5	9.860	155.4	0.0	-4.5	0.0	-0.3	0.0	-2.3	148.3	155.3	-5.2	-0.4	0.0	149.6
AB1	4.777	-181.4	34.9	-46.4	26.3	-10.8	2.9	-17.2	-191.8	-180.7	-30.0	-6.6	23.4	-193.8
AB2	4.731	-153.6	27.2	-38.7	22.2	-8.8	2.3	-14.7	-164.0	-152.8	-25.2	-4.9	17.5	-165.4
AB3	6.584	-119.5	3.9	-10.7	3.9	-2.0	0.4	-4.9	-128.9	-118.5	-6.8	-1.3	1.1	-125.4
AB4	4.702	-169.9	19.0	-32.7	18.0	-6.8	1.7	-17.7	-188.4	-169.5	-21.1	-4.3	11.0	-183.8
AB5	4.710	-151.5	30.3	-41.5	21.7	-9.3	2.4	-14.8	-162.8	-145.5	-24.1	-4.4	15.2	-158.8
AB6	6.062	-138.5	14.0	-23.0	11.8	-4.7	1.2	-9.4	-148.6	-137.9	-2.9	-2.9	8.2	-147.4
AB7	4.435	-144.2	9.9	-23.8	15.1	-5.2	1.4	-5.1	-151.8	-141.0	-11.8	-2.9	4.4	-151.3
AB8	3.306	-156.0	13.0	-32.3	22.5	-7.5	2.0	-4.2	-162.5	-151.2	-15.6	-4.1	5.6	-165.4
AB9	6.056	-91.6	0.6	-3.6	0.9	-0.7	0.1	-2.0	-96.4	-92.3	-2.6	-0.3	0.0	-95.1
BB1	3.692	85.6	3.8	-11.6	5.6	-2.9	1.0	-1.5	80.1	89.2	-5.2	-1.2	2.3	-85.1
